# Recent Advances in Aggregation-Induced Electrochemiluminescent Biosensors

**DOI:** 10.3390/bios15080471

**Published:** 2025-07-22

**Authors:** Likang Zhou, Junhao Fei, Suping Zhang, Tianyu Shan

**Affiliations:** 1 College of Advanced Materials Engineering, Jiaxing Nanhu University, Jiaxing 314001, China; zhoulikang@jxnhu.edu.cn (L.Z.); fjh240031@jxnhu.edu.cn (J.F.); 206002@jxnhu.edu.cn (S.Z.); 2 Stoddart Institute of Molecular Science, Department of Chemistry, Zhejiang University, Hangzhou 310058, China

**Keywords:** aggregation-induced emission, biosensors, electrochemiluminescence, luminophores

## Abstract

Electrochemiluminescence (ECL) biosensors based on aggregation-induced emission (AIE) emitters have recently emerged as highly sensitive tools for biosensing. The AIE phenomenon, characterized by a significant luminescence change upon aggregation due to restricted intramolecular rotation or vibration, effectively enhances ECL intensity and efficiency, endowing AIECL emitters with high selectivity and stability. This review provides an overview of the developmental trajectory of AIECL, systematically elaborates and comparatively analyzes the mechanisms and luminophore systems of conventional ECL and AIECL, discusses the design strategies and construction methods of AIECL luminophores, and comprehensively summarizes the innovative applications of AIECL in the realm of biosensors. Finally, some of the current challenges in this emerging field are outlined, along with perspectives on future trends.

## 1. Introduction

Biomolecules, as core participants in life activities, not only regulate organismal metabolism and functional homeostasis but also serve as critical biomarkers for disease diagnosis [1,2,3,4,5,6,7,8]. Precise detection of their concentration variations holds paramount significance for early screening of major diseases and personalized therapeutic interventions. Conventional detection methods are often constrained by inadequate sensitivity and operational complexity, driving the continuous exploration of novel analytical techniques [9,10,11,12,13,14,15,16]. Biosensors have emerged as pivotal tools in biomedical detection owing to their rapid response, high specificity, and facile miniaturization [17,18,19,20,21,22,23]. Chemiluminescence (CL), as a cornerstone of optical biosensing, generates light through exergonic chemical reactions without external excitation sources. This unique characteristic enables near-zero optical background, high signal-to-noise ratios, and simple instrumentation, making CL a powerful platform for bioanalysis. Diverse CL systems—including 1,2-dioxetane, peroxyoxalate, and bioluminescence—have been extensively developed for detecting biomolecules [24]. Electrochemiluminescence (ECL), a specialized CL modality, triggers luminescence via electrochemical reactions at electrode surfaces. This approach inherits the near-background-free advantage of CL while offering enhanced spatiotemporal controllability and tunable reaction kinetics [25,26,27,28,29,30,31]. ECL biosensors integrate biomolecules (e.g., antibodies, peptides, nucleic acids, enzymes) as biorecognition elements with electrochemical transduction systems, establishing integrated detection platforms that convert molecular-specific recognition into quantifiable ECL signals [32,33,34,35,36,37,38]. As hybrid systems combining ECL technology with biosensors, ECL biosensors inherently possess both the high sensitivity of ECL and the exceptional specificity of biosensors [39,40,41,42,43,44]. Currently, ECL biosensors have been successfully implemented in bioassays targeting diverse analytes, including inorganic substances [45,46,47,48], organic small molecules [49,50,51,52,53], proteins [54,55,56,57], nucleic acids [58,59,60,61,62,63,64], and cells [65,66,67,68,69,70,71,72,73,74,75,76,77,78]. Nevertheless, the performance of ECL biosensor platforms is fundamentally dictated by the properties of luminophores, which serve as critical components. Conventional ECL luminophores typically suffer from aggregation-caused quenching (ACQ) effects, where strong π–π stacking interactions and excitonic energy transfer in aggregated states promote non-radiative decay pathways, drastically reducing emission efficiency. This phenomenon, combined with low emission efficiency in aqueous/aggregated states, potential biotoxicity, and insufficient photostability, significantly impedes performance optimization and clinical translation of ECL biosensors [79,80]. Notably, alternative strategies exist to achieve intense emission at high concentrations, such as designing molecular crystals with controlled intermolecular charge-transfer interactions. Specific molecular packing creates low-lying intermolecular charge-transfer states that enable efficient exciton migration while suppressing non-radiative decay through restricted molecular rotation. This mechanism significantly enhances solid-state luminescence [81]. Consequently, developing novel ECL luminophores with concurrent high emission efficiency, superior biocompatibility, and environmental stability has emerged as a critical challenge.

The discovery of the aggregation-induced emission (AIE) phenomenon by Benzhong Tang’s team in 2001 provided a breakthrough solution to this dilemma [82]. Traditional luminescent systems frequently exhibit ACQ behavior, where emission occurs primarily under dilute conditions but diminishes or quenches completely upon molecular aggregation at elevated concentrations. Unlike conventional ACQ materials, AIE-active materials display weak emission in molecularly dispersed states but demonstrate significantly enhanced luminescence efficiency in aggregated states through restricted intramolecular motion (RIM) effects that suppress non-radiative decay pathways. This characteristic aligns perfectly with ECL technology’s requirement for solid-state luminophores, opening new avenues for constructing advanced ECL systems.

In 2017, Cola’s team pioneered the concept of aggregation-induced electrochemiluminescence (AIECL) through investigations into the supramolecular nanostructures of square-planar Pt (II) complexes [83]. They observed that Pt (II) complexes exhibit substantially enhanced ECL intensity in aggregated states compared to dispersed states, attributed to modifications in the highest occupied molecular orbital (HOMO)–lowest unoccupied molecular orbital (LUMO) energy gap during self-assembly processes. AIECL materials not only address the inherent limitations of conventional luminophores (e.g., poor aqueous biocompatibility and low solid-state efficiency), but their “aggregation-enhanced” characteristics enable environmental adaptability through controlled molecular packing, simultaneously reducing background noise and improving detection sensitivity [84,85,86,87,88,89,90]. This review systematically elaborates upon the developmental history of AIECL, compares conventional ECL mechanisms and material systems, analyzes the design strategies of AIECL luminescent bodies guided by the AIE mechanism, deeply explores the construction methods of AIECL luminescent bodies, and comprehensively summarizes their innovative applications in small molecule detection, DNA detection, and protein marker analysis. Finally, the future development direction for AIECL biosensors is projected.

## 2. ECL Biosensors

**Figure 1:**
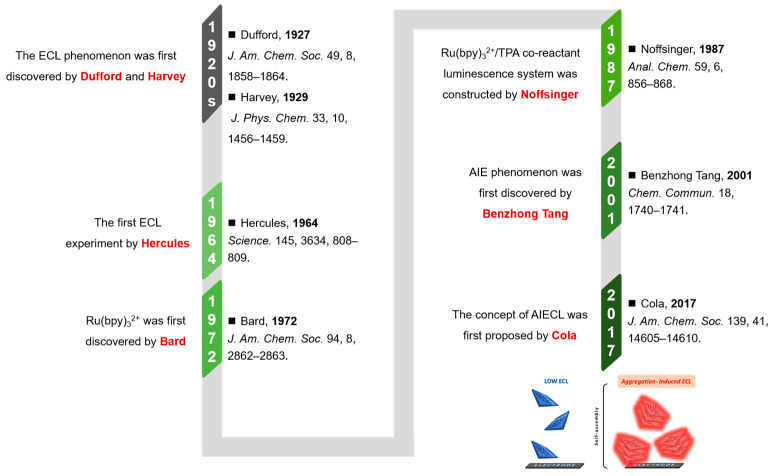
A schematic diagram of the developmental trajectory of AIECL [82,83,91,92,93,94,95]. Reproduced with permission from ref. [83]. Copyright 2017, American Chemical Society.

The ECL phenomenon can be traced back to the 1920s. As illustrated in Figure 1, Dufford et al. first observed the electrolytic luminescence of Grignard reagents in 1927 [91], followed by Harvey’s report on anodic electrochemiluminescence behavior in alkaline luminol solutions in 1929 [92]. Constrained by early analytical techniques, ECL research remained stagnant until 1964, when Hercules’ team first reported the cathodic electrochemiluminescence of aromatic hydrocarbons in organic solvents [93], marking the inception of modern ECL studies. In 1972, Bard’s research group identified ruthenium trisbipyridine (Ru(bpy)_3_^2+^) as a landmark ECL luminophore with exceptional performance [94], whose high quantum efficiency and chemical stability established it as the most widely utilized electrochemiluminescent probe molecule. Noffsinger developed the Ru(bpy)_3_^2+^/tripropylamine (TPA) coreactant luminescence system in 1987, significantly enhancing ECL detection sensitivity [95]. The inaugural application of ECL technology in biodetection in 1989 formally initiated its rapid advancement in biosensing. Current research focuses on developing novel luminophores, optimizing reaction kinetics, and constructing multifunctional integrated sensing platforms to meet biosensing requirements for high sensitivity, stability, and low biotoxicity.

### 2.1. Mechanism of ECL

Electrochemiluminescence is a luminescent phenomenon arising from the redox reactions of luminophores at electrode surfaces, where excited-state intermediates emit photons upon returning to their ground state. Fundamentally, ECL involves high-energy electron transfer at the working electrode surface under applied electrochemical waveforms, driving multi-step reactions between luminophores and coreactants to generate excited states and subsequent photon emissions. Compared to conventional chemiluminescence (e.g., thermochemiluminescence systems that utilize temperature-triggered reactions for spatiotemporal control) [96,97], ECL retains distinct advantages through electrochemical excitation mechanisms: (1) spatiotemporal controllability: precise regulation of reaction initiation/termination via electrode potential modulation, differing from thermochemiluminescence that relies on concentration, temperature, etc.; (2) near-zero background interference: electrochemical excitation eliminates stray light from external sources; and (3) ultrahigh sensitivity: single-electron-transfer processes enable detection at single-molecule levels. These attributes endow ECL with broad application prospects in clinical diagnostics, environmental monitoring, and food-safety analysis.

ECL mechanisms are primarily classified into annihilation-type and coreactant-type systems. Annihilation-type ECL requires alternating oxidation/reduction potentials to generate luminophore cations (L^+^) and anions (L^−^), which undergo electron-transfer annihilation to form excited states (L) for light emission, exemplified by polycyclic aromatic hydrocarbons [98,99,100,101,102]. Coreactant-type ECL employs sacrificial reagents (e.g., TPA, persulfate) whose oxidized/reduced intermediates cascade-react with luminophores: in the Ru(bpy)_3_^2+^/TPA system, TPA is anodically oxidized to TPA^+^ cations, which spontaneously deprotonate into highly reductive TPA species that reduce Ru(bpy)_3_^3+^ to excited-state Ru(bpy)_3_^2+^ for emission. The coreactant mechanism, obviating bipolar potential switching, demonstrates superior compatibility with biosensing system architectures [94,103].

### 2.2. ECL Luminophores

ECL biosensors comprise three core components: (1) biorecognition elements (e.g., antibodies, peptides, nucleic acids, enzymes) that enable target capture via specific binding interactions; (2) ECL luminophores serving as signal transducers, whose redox activity and luminescence efficiency dictate detection performance; and (3) electrode substrates (e.g., gold, glassy carbon, or ITO-coated electrodes) whose surface modifications govern electron-transfer efficiency and biomolecule immobilization efficacy. ECL-based biosensors employ biosensitive materials (e.g., antibodies, peptides, nucleic acids, enzymes) as recognition units to specifically identify targets, converting bioaffinity interactions into quantifiable ECL signals through a sensing interface and transduction system for target concentration quantification.

As energy conversion hubs, luminophores undergo redox processes in ECL systems to generate metastable excited-state intermediates, releasing energy as photons detected by photodetection instrumentation. Their intrinsic properties—emission wavelength, spectral full width at half maximum (FWHM), and luminescence quantum yield—directly determine sensor sensitivity and selectivity [104]. Consequently, ECL advancements are intrinsically linked to luminophore innovation. The evolution of ECL biosensors has driven the expanded diversity of luminophores, now categorized into inorganic luminophores, organic luminophores, and nanomaterial-based emitters.

#### 2.2.1. Inorganic Luminophores

Inorganic ECL luminophores primarily rely on metal complex systems, where luminescent architectures are constructed via coordination interactions between metal ions and ligands, encompassing complexes of Ru, Os, Cr, Cd, Pd, Pt, Re, Ir, Mo, Tb, Eu, and Cu. Ruthenium complexes dominate this field due to their unique photophysical properties. Tris(2,2′-bipyridine)ruthenium(II) (Ru(bpy)_3_^2+^), the most extensively studied ECL luminophore, exhibits three key merits: (1) excellent aqueous stability, high luminescence efficiency, and favorable electrochemical properties; (2) superior solubility in both aqueous and non-aqueous media enabling broad solvent compatibility; and (3) reversible electrochemical behavior and well-understood emission mechanisms [105]. Its emission mechanism operates through either annihilation or coreactant pathways, demonstrating exceptional compatibility with diverse coreactants to expand application scopes [106,107,108,109,110]. The Ru(bpy)_3_^2+^/TPA coreactant system achieves superior ECL emission, with detection limits as low as pmolar levels and linear ranges spanning six orders of magnitude. These attributes establish Ru(bpy)_3_^2+^ as a cornerstone of both fundamental research and commercial applications, particularly in clinical ECL immunoassays and DNA detection platforms.

**Figure 2:**
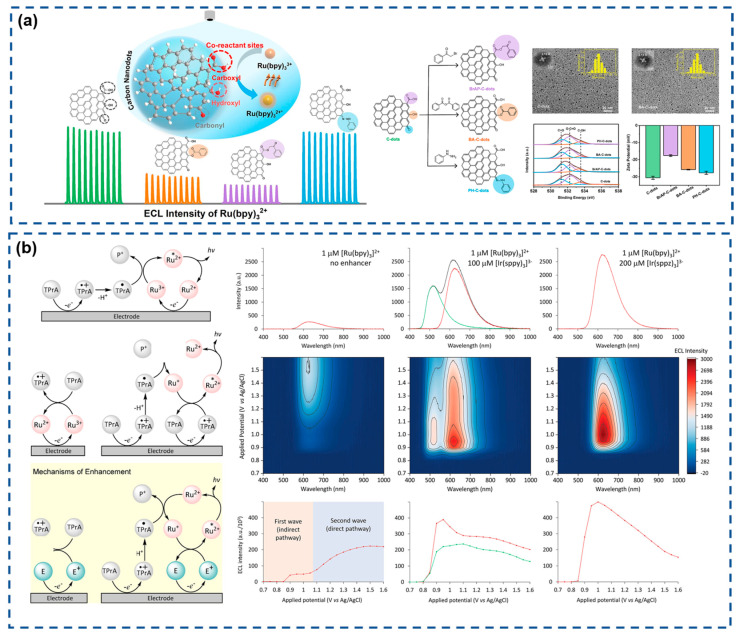
(**a**) Schematic diagram of the enhanced effect of selective deactivation of carboxyl, hydroxyl, and carbonyl groups on the ECL phenomenon at C-dots. Reproduced with permission from ref. [111]. Copyright 2023, Elsevier. (**b**) Reaction pathways for the coreactant ECL of Ru(bpy)_3_^2+^/TPA and ECL spectra. Reproduced with permission from ref. [112]. Copyright 2024, Wiley-VCH.

Recent advances have focused on optimizing luminophore systems to amplify ECL signals, driven by increasing demands for rapid and ultrasensitive biomarker quantification. Peng et al. (Figure 2a) engineered oxygen-containing functional groups on carbon nanodots (CNDs) via chemical modification to investigate their role as coreactants in Ru(bpy)_3_^2+^-based ECL systems [111]. The selective deactivation of carboxyl, hydroxyl, and carbonyl groups on CNDs revealed carboxyl groups as pivotal mediators in the ECL process. Hydrogen peroxide-mediated carboxyl enrichment enhanced ECL intensity by 10-fold compared to pristine systems, offering novel insights for designing Ru(bpy)_3_^2+^-based coreactant architectures. Francis et al. (Figure 2b) innovatively developed a non-emissive enhancer [Ir(sppz)_3_]^3−^ that amplifies ECL signals in Ru(bpy)_3_^2+^/TPA systems [112]. Unlike conventional enhancers, [Ir(sppz)_3_]^3−^ operates without self-emission, allowing high-concentration utilization for 11-fold signal amplification without spectral interference. This enhancer boosts ECL through electrocatalytic TPA oxidation and efficient chemical excitation of Ru(bpy)_3_^2+^ emitters. This approach presents a promising strategy for sensitivity enhancement without requiring sophisticated instrumentation.

**Figure 3:**
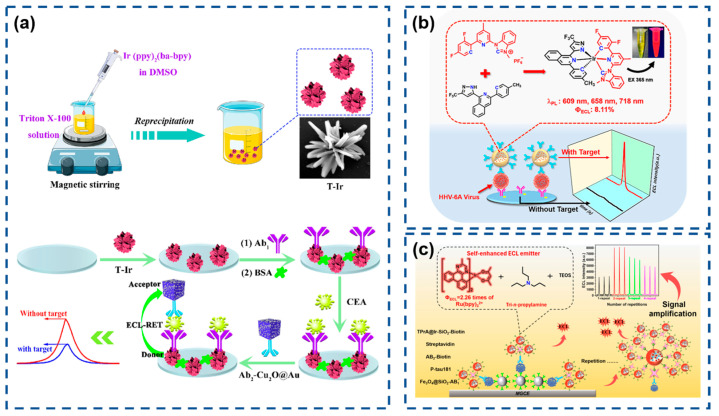
(**a**) Schematic illustration of the preparation of T-Ir and Ab2-Cu_2_O@Au and the construction of the ECL immunosensor for detection. Reproduced with permission from ref. [115]. Copyright 2023, Elsevier. (**b**) Preparation of BisLT-Ir-NHC and schematic representation of the highly efficient immunosensor. Reproduced with permission from ref. [116]. Copyright 2024, American Chemical Society. (**c**) Synthetic for Ir(mdq)_2_(acac) and preparation of TPrA@Ir-SiO_2_ and ultrasensitive ECL magnetic immunosensor. Reproduced with permission from ref. [117]. Copyright 2025, American Chemical Society.

Iridium(III) complexes have emerged as attractive alternatives for novel ECL luminophores due to their straightforward synthesis, high quantum yields, and extended emission lifetimes. Compared to Ru(III) complexes, Ir(III) systems exhibit stronger ligand field splitting energies derived from metal–ligand delocalized HOMOs, resulting in superior quantum efficiencies [113,114]. However, their development in ECL applications has been hindered by poor aqueous solubility and oxygen-induced quenching. Addressing these limitations, Song et al. (Figure 3a) synthesized hydrophilic iridium(III) nanoflowers (T-Ir) via nonionic surfactant-assisted reprecipitation, demonstrating strong ECL activity at low potentials (−1.10 V) with potassium persulfate as a coreactant [115]. Integration with gold nanoparticle-decorated cuprous oxide nanocubes (Cu_2_O@Au) as quenchers enabled the construction of a T-Ir-based ECL biosensor for the labeled detection of antibodies (Ab_2_). Dai’s group (Figure 3b) developed bis-tridentate Ir(III) complexes (BisLT-Ir-NHC) with N-heterocyclic carbene (NHC) ligands, encapsulated within silica nanoparticles to mitigate solubility constraints [116]. Coupled with MXene-modified sensing interfaces, this system established a sensitive sandwich immunosensor with electrochemical properties validated by cyclic voltammetry. As shown in Figure 3c, further innovation produced self-enhanced nanoemitters (TPrA@Ir-SiO_2_) by co-encapsulating Ir(mdq)_2_(acac) luminophores and coreactants within silica nanoparticles, confining coreactant diffusion distances to the nanoscale via spatial confinement effects [117]. Subsequent signal amplification was achieved through streptavidin–biotin high-affinity interactions.

#### 2.2.2. Organic Luminophores

Luminol has become the most studied organic ECL luminophore due to its non-toxicity, excellent aqueous solubility, low oxidation potential, and minimal background interference [118,119]. While other organic systems (e.g., 2-coumaranones) exhibit ECL activity, their mechanistic understanding and biosensing applications remain underexplored compared to established luminophores. Luminol-based ECL systems are primarily categorized into cathodic luminol ECL and anodic luminol ECL. Cathodic luminol ECL relies on electrochemically reduced dissolved oxygen to generate reactive oxygen species (ROS), yet its intensity remains inherently weak due to low oxygen solubility and luminol’s electrochemical inactivity at negative potentials. Anodic luminol ECL employs alkaline hydrogen peroxide (H_2_O_2_) as a coreactant to amplify emission intensity through synergistic reaction mechanisms. The biological relevance of H_2_O_2_ in metabolic processes underpins its widespread adoption in biosensing applications [120,121,122,123]. However, limitations, including H_2_O_2_’s self-decomposition and low dissolved oxygen concentrations, hinder further development, necessitating novel coreactant systems to overcome performance bottlenecks.

**Figure 4:**
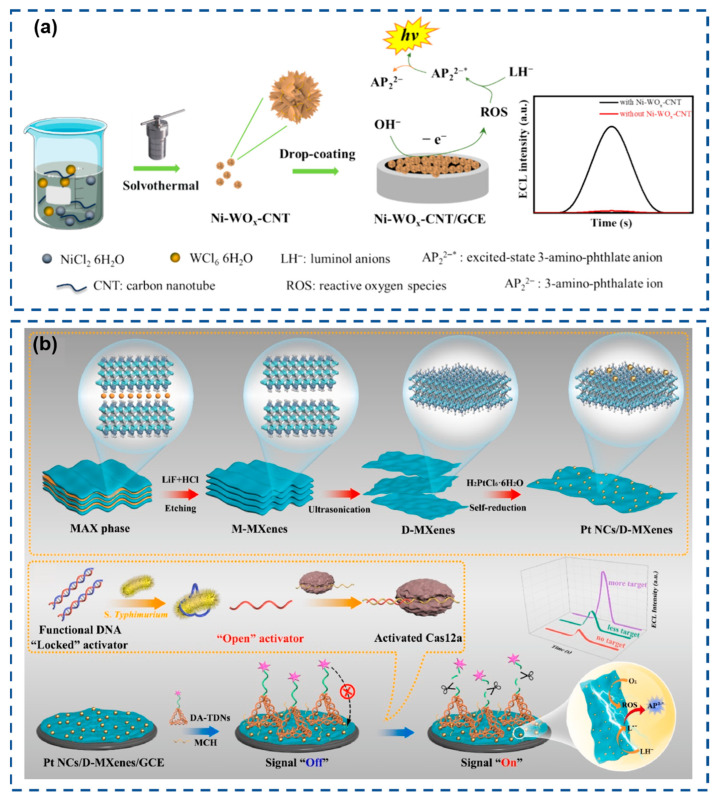
(**a**) Schematic illustration of constructing a luminol–OH^–^ ECL system using Ni-WOx-CNT as a coreactant accelerator. Reproduced with permission from ref. [124]. Copyright 2024, American Chemical Society. (**b**) Schematic illustration of the synthesis of Pt NCs/D-MXenes and the sensor. Reproduced with permission from ref. [125]. Copyright 2025, Elsevier.

To address these challenges, Li et al. (Figure 4a) innovatively proposed hydroxide ions (OH^−^) as alternative coreactants, synthesizing nickel-doped carbon nanotube-modified tungsten oxide (Ni-WOx-CNT) via a solvothermal method to function as a coreactant accelerator [124]. Ni-WOx-CNT exhibits exceptional catalytic activity, generating abundant ROS from OH^−^ at low excitation potentials to drive sustained luminol ECL emission, thereby establishing a robust luminol-OH^−^-(Ni-WOx) ECL platform. Cao et al. (Figure 4b) engineered ultrafine platinum nanoclusters anchored on two-dimensional hierarchical MXenes (Pt NCs/D-MXenes) as coreactant accelerators for luminol-O_2_ ECL systems [125]. The ultrathin D-MXenes substrate modulates Pt NCs’ size/dispersion and exposes active sites, while synergistic D-MXenes/Pt NC interactions enhance oxygen reduction electrocatalysis, boosting ROS generation for intensified ECL emission with superior catalytic efficiency.

#### 2.2.3. Luminescent Nanoparticles

In 2002, Bard’s research group first reported quenching-type and coreactant-type ECL phenomena in silicon quantum dots [126], while subsequent studies by Ding’s team on silicon nanocrystals sparked widespread interest in nanomaterial-based ECL systems [127,128,129]. Numerous nanostructures have demonstrated ECL activity, including quantum dots (QDs), metal nanoclusters (Ag, Au, Cu), carbon dots, metal–organic frameworks (MOFs), layered materials, and semiconductor nanocrystals (e.g., CdSe, CdTe, ZnSe). The surface structure and morphology of nanomaterials critically influence their ECL performance, driving current research to focus on nanoscale luminophore engineering.

**Figure 5:**
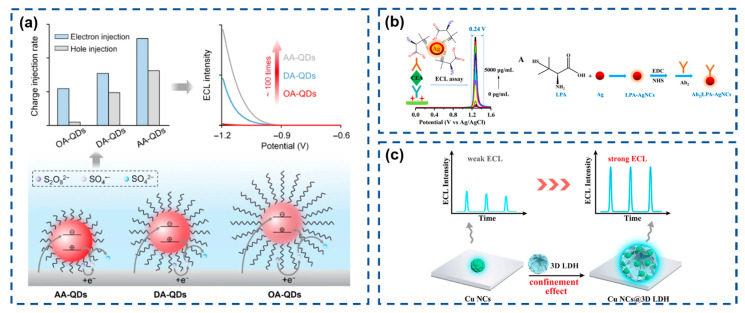
(**a**) Schematic diagrams of the CdSe/CdS/ZnS core/shell/shell QDs with different organic ligands and the effects on ECL performance. Reproduced with permission from ref. [130]. Copyright 2024, Springer Nature. (**b**) Schematic illustration for the bioconjugates and narrow-triggering-potential-window ECL immunosensor. Reproduced with permission from ref. [131]. Copyright 2024, American Chemical Society. (**c**) Schematic diagrams of the preparation of CuNCs@3D-LDH and the effects on ECL performance. Reproduced with permission from ref. [132]. Copyright 2024, Elsevier.

QDs, as emerging ECL emitters, surpass conventional molecular probes in both emission efficiency and signal modulation capability, making their integration into efficient ECL systems scientifically significant. However, QD emission is highly susceptible to surface defects. While core/shell architectures or surface ligands can passivate defect states, wide-bandgap shells and inert ligands inevitably impede carrier injection, compromising ECL efficiency. Su’s team (Figure 5a) systematically investigated how surface ligands affect the ECL performance of CdSe/CdS/ZnS core/shell/shell QDs [130]. Results revealed that shortening ligand chains substantially enhances electron/hole injection rate constants in QD films, thereby optimizing ECL characteristics.

Continuous innovation in nano-ECL systems propels technological advancements in this field. Gao et al. (Figure 5b) developed a silver-based ECL emitter using L-penicillamine-functionalized Ag nanoclusters (LPA-Ag NCs), which demonstrate efficient redox-mediated ECL with a narrow trigger potential window (0.24 V) when paired with a hydrazine (N_2_H_4_) coreactant [131]. Yuan’s group (Figure 5c) engineered copper nanoclusters confined within 3D flower-like layered double hydroxide (Cu NCs@3D-LDH), leveraging its high surface area and hierarchical morphology [132]. This architecture simultaneously increases the local Cu NC concentration to enhance emission intensity and restricts intramolecular motion to suppress non-radiative transitions, achieving remarkable ECL amplification.

## 3. AIECL Biosensors

### 3.1. Mechanism of AIEs

**Figure 6:**
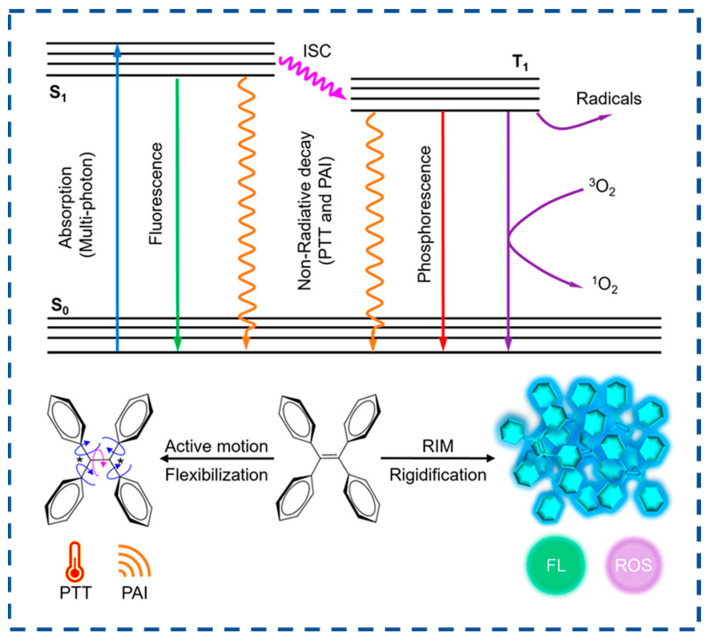
The molecular mechanism of the AIE phenomenon (reproduced with permission from ref. [134]. Copyright 2023, American Chemical Society).

AIEs are a unique class of photophysical phenomena characterized by intense emission upon aggregation while remaining non-emissive in dilute solutions. Over the past two decades of AIE research, the working mechanism of RIM has been well-accepted [133,134]. In the solution state, vigorous molecular motions serve as relaxation channels that facilitate non-radiative decay of excited states. In contrast, in the aggregation state, these molecular motions are significantly suppressed due to spatial constraints, thereby blocking non-radiative decay pathways and promoting the radiative decay of excited states (Figure 6) [134]. Compared to conventional fluorescent materials, AIE materials have low background noise, high luminescence efficiency, high biocompatibility, high photostability, and structural diversity.

### 3.2. AIECL Luminophores

Recently, many AIE-based ECL luminophores have been studied, opening a new pathway for developing high-performance ECL sensors. Based on their structural characteristics, these luminophores can be classified into three major categories: metal clusters, low-molecular-weight AIE molecules, and polymeric AIE molecules.

**Figure 7:**
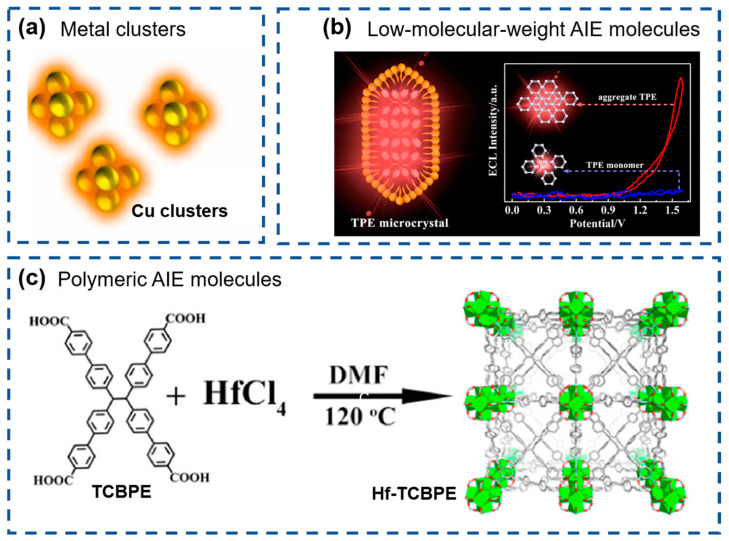
Three major categories of AIECL luminophores: (**a**) metal clusters (reproduced with permission from ref. [135]. Copyright 2022, Elsevier), (**b**) low-molecular-weight AIE molecules (reproduced with permission from ref. [139]. Copyright 2019, American Chemical Society), and (**c**) polymeric AIE molecules (reproduced with permission from ref. [140]. Copyright 2020, American Chemical Society).

Metal clusters (Figure 7a) can exhibit AIE characteristics due to their unique quantum confinement effect [135] and have gained traction as ECL emitters due to their straightforward synthesis, excellent biocompatibility, and tunable optical properties, etc. [136]. Noble metal clusters, particularly gold nanoclusters (Au NCs) and silver nanoclusters (Ag NCs), have been comprehensively studied for ECL applications [137]. Compared to noble metal clusters, non-noble metal clusters are more cost-effective and have garnered significant attention in recent years, as reported for Cu, Zn NCs, et al. [138].

Low-molecular-weight AIE molecules (Figure 7b), particularly TPE derivatives, have been widely employed in ECL applications owing to their strong emission upon aggregation in aqueous environments, which is highly advantageous for biosensing [139]. Recent studies have demonstrated that micro- or nano-crystals exhibit enhanced ECL emission, as lattice constraints effectively suppress intramolecular rotations and vibrations, thereby reducing non-radiative energy dissipation. Moreover, the ordered crystalline structure of microcrystals promotes efficient energy transfer and facilitates the generation of excited states to produce stable and intense luminescent signals. For example, Jiang et al. reported a significantly enhanced ECL of TPE microcrystals than of their monomeric counterparts due to restricted intramolecular motions [139].

Polymeric AIE molecules (Figure 7c), characterized by their high molecular weight, represent another important class of ECL-active materials [140]. These polymers inherently possess AIE properties conferred by functional moieties, such as TPE and anthracene units, etc. To further amplify the ECL performance, metal ion coordination has been employed to effect metal-to-ligand charge transfer (MLCT) and generate excited states, including metal-coordination polymers [141] and MOFs [142,143]. Particularly, as a representative of emerging materials, MOFs received considerable attention due to their high specific surface area, large pore size and high stability. Xiao et al. demonstrated that Hf-TCBPE exhibited a stronger ECL emission than the corresponding ligand aggregates [140]. The strong ECL intensity of Hf-TCBPE originated from the rigid immobilization of TCBPE ligands and the high porosity of Hf-TCBPE, collectively enabling the efficient excitation of both internal and external chromophores.

### 3.3. Construction Methods of AIECL Biosensors

AIECL characterizes a scenario in which molecular assemblies demonstrate a marked increase in electrochemiluminescence quantum yield upon supramolecular organization. The photophysical characteristics of luminescent materials serve as a critical determinant for the detection limit and signal-to-noise ratio in sensor systems [144]. Deciphering the structural-property correlations governing luminophore architectures is pivotal for engineering biosensing platforms with tailored biorecognition capabilities [84].

**Figure 8:**
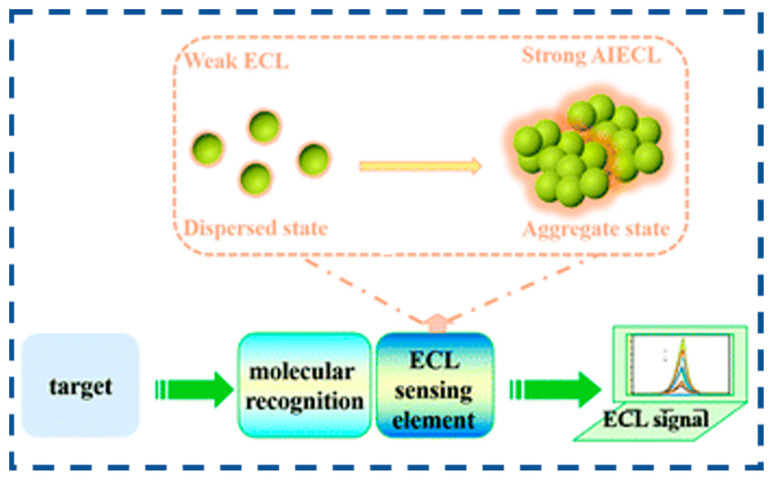
Schematic diagram of the principle of the AIECL biosensors (reproduced with permission from ref. [85]. Copyright 2022, Royal Society of Chemistry).

AIECL materials can exist in various forms, including simple molecules or polymers, as well as more intricate supramolecular structures and porous frameworks, like MOFs and COFs. As shown in Figure 8, through meticulous molecular engineering, the frontier molecular orbitals, electronic transitions, and exciton utilization efficiency can be precisely tuned to achieve optimal luminescent performance. In their aggregated state, AIECL materials typically enhance luminescence by RIM, which encompasses limiting intramolecular rotation (RIR) and intramolecular vibration (RIV). By modulating the weak intermolecular interactions, such as π–π stacking, hydrogen bonding, and van der Waals forces, the energy transfer, electron transport, and excited-state lifetime can be optimized to fulfill specific functional requirements. With thoughtful molecular design and the selection of suitable building blocks, the macro- and microstructures of AIECL materials can be precisely controlled. This enables the adjustment of their luminescent properties for tailored functional applications. The typical construction strategies for AIECL biosensors are outlined below.

#### 3.3.1. Structure Design of Enzymes

**Figure 9:**
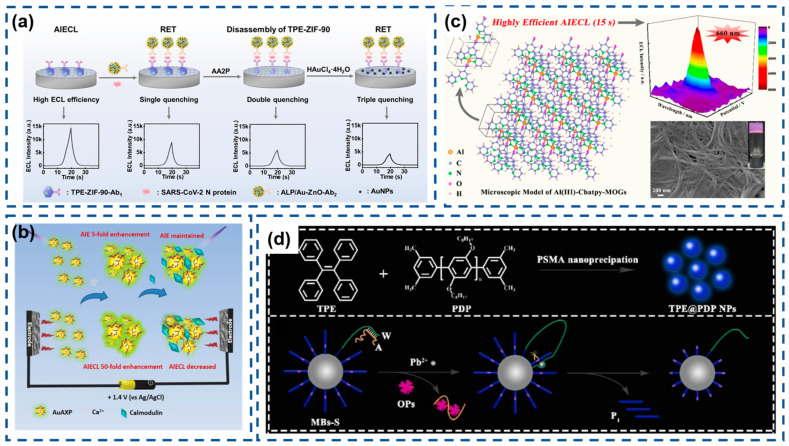
(**a**) AIECL probe based on enzyme substrate. Reproduced with permission from ref. [145]. Copyright 2024, American Chemical Society. (**b**) Construction of an AIECL sensor for calmodulin detection. Reproduced with permission from ref. [147]. Copyright 2019, Wiley-VCH. (**c**) Self-assembling metal-organic gel with significant AIECL characteristics. Reproduced with permission from ref. [148]. Copyright 2022, American Chemical Society. (**d**) Encapsulation-modulated aggregation strategy with a large π−conjugated polymer. Reproduced with permission from ref. [149]. Copyright 2024, Elsevier.

Enzymes, commonly found throughout living systems, demonstrate exceptional molecular recognition capabilities and remarkable reaction acceleration properties. These characteristics make them ideal diagnostic indicators and therapeutic agents in medical applications. Specifically, peptide bond cleavage mediated by these biocatalysts can induce AIECL molecular assembly. The construction of AIECL bioprobes typically commences with aggregation strategies triggered by three distinct reactions: enzyme-triggered precipitate, enzyme-catalyzed coupling, and enzyme-instructed self-assembly (Figure 9a) [145]. Very recently, Ma et al. reported an amphiphilic anionic platinum(II) bzimpy complex PS-BZIMPY-Pt with the AIECL property [146]. Its aggregate reveals intense anodic and cathodic ECL emissions. Due to the suppressed formation of sulfate radicals (SO4•) by glutathione (GSH) and glutathione reductase (GR) during the cathodic ECL process, which quenches the ECL emission of PS-BZIMPY-Pt, an ECL sensor for detecting GSH was developed with a broad linear detection range of 0.1–200 μM and a low limit of detection of 0.016 μM.

#### 3.3.2. Ligand–Receptor Specific Interaction

The research team led by Jiang developed an innovative hydrogel system constructed through the supramolecular assembly of gold nanoclusters cross-linked by divalent metal cations (Ca^2+^, Mg^2+^, and Zn^2+^). The hydrogel demonstrates dual functionality through both AIE and AIECL phenomena. Notably, the calcium-binding protein calmodulin was found to effectively modulate AIECL responses via specific interactions with the Ca^2+^ cross-linking moieties. Quantitative analysis revealed a linear detection range spanning 0.3-50 μg mL^−1^ for calmodulin detection, with an impressive detection limit of 0.1 μg mL^−1^. Most remarkably, the system exhibited substantially greater enhancement in AIECL performance (~50-fold intensity increase) compared to its AIE response (~5-fold enhancement), highlighting the exceptional electrochemiluminescent amplification capability of this nanocluster-based hydrogel architecture (Figure 9b) [147].

#### 3.3.3. Self-Assembly

AIECL initially originated from the self-assembly behavior of square-planar Pt (II) [83]. In recent work by Han and colleagues, an innovative aluminum(III)-carboxylated terpyridine metal-organic gel (Al(III)-Cbatpy-MOG) system was engineered through ultrafast coordination (<15 s) between Al^3+^ ions and 4′-carboxylic acid-2,2′:6′,2″-terpyridine (Cbatpy) ligands. The resulting nanostructured material exhibits exceptional AIECL enhancement, substantially outperforming conventional ECL-active supramolecular gels. The rapid self-assembly mechanism leverages strong N,O-chelating interactions between Cbatpy’s heteroatoms and Al^3+^ cations, enabling the single-step fabrication of highly viscous, stable MOGs with nanofibrillar architecture. This advancement effectively addresses two persistent limitations in AIECL emitter development: cumbersome synthetic procedures and inadequate film-forming properties. Remarkably, the superemissive Al(III)-Cbatpy-MOG system demonstrates a 20-fold enhancement in ECL quantum yield compared to discrete Cbatpy molecules, a phenomenon ascribed to the aggregation-mediated suppression of the intramolecular rotational/torsional motions that typically promote nonradiative decay pathways (Figure 9c) [148].

#### 3.3.4. Microencapsulation Encapsulation

Despite their structural tunability and exceptional optoelectronic characteristics, tetraphenylethylene (TPE) derivatives continue to face significant limitations in achieving high ECL quantum yields. π-Conjugated macromolecules can efficiently encapsulate AIE molecules, such as TPE, through hydrophobic interactions. A breakthrough encapsulation-modulated aggregation strategy has recently emerged to amplify the ECL performance of TPE systems. He et al. innovatively employed poly [2,5-dioctyl-1,4-phenylene] (PDP) as a supramolecular host matrix, leveraging its hydrophobic properties to achieve controlled encapsulation of TPE luminophores [149]. The resulting complex TPE@PDP nanoparticles (NPs) not only restricted the molecule motion of TPE due to space limitation but also inhibited the aggregation-caused quenching effect caused by TPE crystals featuring a dense aggregation structure. Crucially, the strong electronic synergy between the PDP framework and confined TPE assemblies facilitated efficient charge carrier migration within the nanostructured composite, ultimately generating intense aggregation-induced AIECL emission with a substantially enhanced quantum yield (Figure 9d).

### 3.4. Applications of AIECL Biosensors

Biosensing platforms utilizing AIECL have demonstrated exceptional target recognition capabilities through multiple molecular interaction mechanisms, including hydrogen bonding networks and hydrophobic interactions. The subsequent discussion highlights representative implementations of AIECL biosensors that have achieved breakthroughs in bioanalytical applications.

#### 3.4.1. Small Molecule Detection

Dopamine, glucose, uric acid, and various other small-molecule compounds serve as crucial raw materials and products in life cycles and metabolic processes. Consequently, numerous biosensors utilizing AIECL materials have been reported for the detection of these small molecules.

As a vital neurotransmitter, dopamine (DA) plays critical roles in the pathogenesis of neurodegenerative and neuropsychiatric disorders, including Parkinson’s disease, schizophrenia, and Huntington’s disease. The quantitative analysis of DA levels has emerged as a critical biomarker for facilitating early-stage diagnostic protocols, monitoring disease progression, and guiding personalized therapeutic interventions [150,151]. A groundbreaking methodology was pioneered by Luo’s research team, involving the synthesis of Ti3C2Tx MXene-stabilized iridium(III) complexes ([Ir(pbi)_2_(acac)]) exhibiting AIECL within a polyvinyl alcohol (PVA) hydrogel matrix. The MXene nanosheets, with their expansive electroactive surface area, enhanced electron-transfer efficiency and amplified the ECL signal intensity of the immobilized iridium complex (Ir@MXene). Subsequent encapsulation of Ir@MXene into the PVA hydrogel (Ir@MXene-PVA) achieved dual functional advantages: (1) spatial confinement elevating the effective concentration of luminophores while blocking oxidative species infiltration, and (2) suppression of molecular vibrations to minimize non-radiative energy loss. This synergistic confinement effect resulted in amplified AIECL emission.

**Figure 10:**
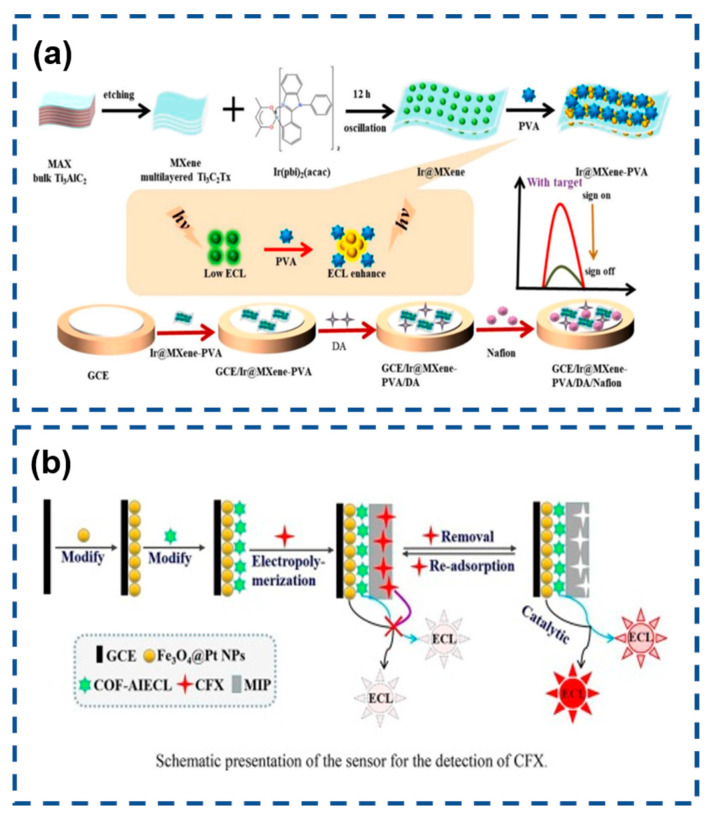
(**a**) Dopamine detection in polymer hydrogel system. Reproduced with permission from ref. [152]. Copyright 2024, Elsevier. (**b**) AIECL molecularly imprinted sensor for CFX monitoring. Reproduced with permission from ref. [155]. Copyright 2022, Elsevier.

Capitalizing on the DA concentration-dependent ECL quenching mechanism, the team engineered a biosensing platform capable of detecting DA in human serum with high sensitivity. The sensor demonstrated a broad linear response from 0.01 to 100 nmol/mL (10 nM–100 µM) and an ultralow detection limit of 2.0 pmol/mL (2.0 nM), surpassing conventional detection thresholds. This innovative strategy establishes a robust framework for designing MXene-integrated luminescent hydrogels, offering significant potential for advancing neurochemical diagnostics (Figure 10a) [152].

Ciprofloxacin (CFX), a third-generation fluoroquinolone antibiotic, exhibits potent bactericidal properties against Gram-positive and Gram-negative pathogens through DNA gyrase inhibition. Beyond its clinical efficacy, CFX demonstrates extended antimicrobial coverage, making it indispensable for prophylactic and therapeutic interventions in both human medicine and veterinary practice. However, its classification as a zoonotic pharmaceutical agent raises significant public health concerns—persistent CFX residues in animal-derived products accumulate through bioamplification in food chains, potentially inducing antibiotic resistance and compromising therapeutic effectiveness in human populations. Given its dual veterinary and clinical applications, the development of advanced analytical platforms capable of detecting trace-level CFX residues (<μg/kg range) in meat, dairy, and aquatic products has become a critical priority for food safety protocols [153,154]. Leveraging covalent organic frameworks (COFs) with intrinsic AIECL properties, Li et al. engineered luminophore-integrated COF-AIECL nanostructures via boric acid-mediated polycondensation. The synergistic integration of AIECL and nanozymatic amplification yielded dual signal modulation: Fe_3_O_4_@Pt NPs catalytically boosted the COF-AIECL emission intensity through peroxidase-mimetic activity, while CFX binding induced steric hindrance and electron-transfer blocking, producing concentration-dependent ECL quenching. This dual-signal engineering strategy achieved remarkable sensitivity enhancements (128-fold vs. conventional ECL systems) alongside molecular imprinting-driven specificity. Optimized detection parameters demonstrated a broad dynamic range (2 fM–3 nM) with an ultra-low limit of detection (598 aM), surpassing existing CFX sensing platforms. Practical validation in complex milk matrices showed excellent recovery rates (92–111%) without pretreatment, confirming robustness against matrix interference (Figure 10b) [155].

#### 3.4.2. DNA and RNA Detection

There are several forms of DNA and RNA in living organisms [156]. While DNA exhibits limited structural polymorphism, RNA demonstrates exceptional architectural versatility within biological systems. Beyond their canonical roles in genetic translation, RNA molecules possess catalytic functions (ribozymes), epigenetic regulatory capacity, and intrinsic genetic coding properties. Notably, dysregulated RNA pathways are implicated in oncogenesis, modulating apoptosis resistance, telomere maintenance, and tumorigenic signaling cascades. These multifunctional attributes establish RNA as a critical diagnostic biomarker, with precise detection methodologies holding transformative potential for clinical theranostics and personalized medicine [157,158].

**Figure 11:**
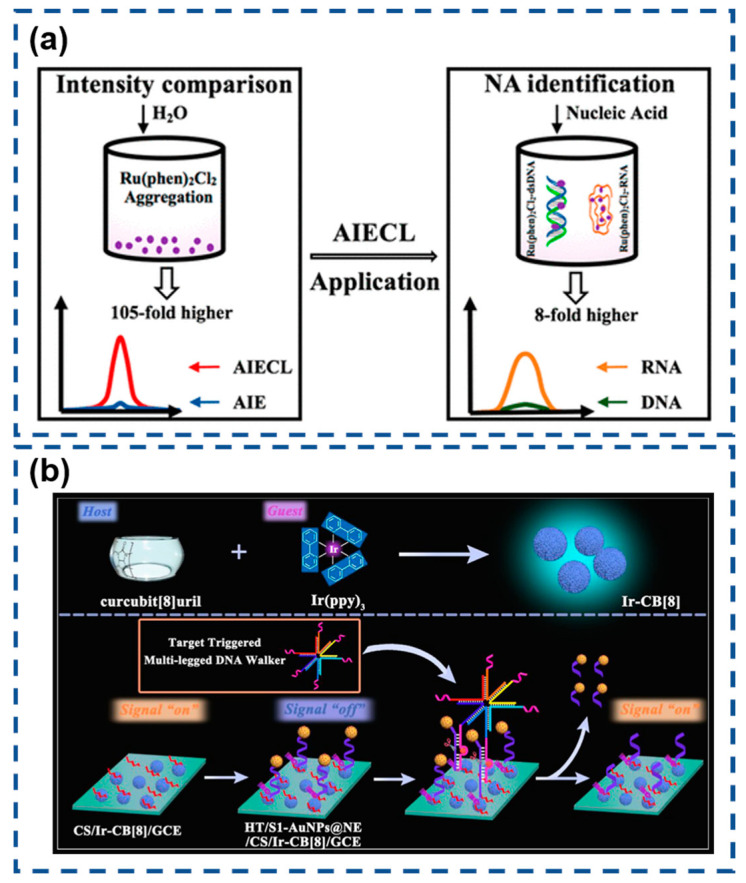
(**a**) AIECL nucleic acid detector in a water–methanol mixed solvent. Reproduced with permission from ref. [159]. Copyright 2024, American Chemical Society. (**b**) Nucleic acid detector featuring host–guest recognition. Reproduced with permission from ref. [160]. Copyright 2024, American Chemical Society.

Recently, Lu et al. conducted a comprehensive study on AIECL using the Ru(phen)_2_Cl_2_/TPrA system in H_2_O-MeCN mixed solvents [159]. Remarkably, increasing the water content from 30% to 70% (*v*/*v*) enhanced the ECL intensity by 120-fold, significantly surpassing the 5.7-fold fluorescence enhancement observed under similar conditions. Advanced characterization techniques confirmed that this dramatic signal amplification originated from the progressive formation of Ru(phen)_2_Cl_2_ nanoaggregates in high-water-content environments. The exceptional sensitivity of this AIECL phenomenon enables precise discrimination between RNA and DNA, as well as among different miRNA species. This breakthrough demonstrates considerable potential for developing next-generation biosensors, particularly in applications requiring ultra-sensitive nucleic acid detection, including cancer diagnostics, subcellular imaging, and real-time biomolecular monitoring (Figure 11a).

Afterwards, Zhao and colleagues pioneered a host–guest recognition strategy for constructing supramolecular AIECL systems, effectively addressing the inherent limitations of conventional electrostatic integration approaches [160]. Their design leveraged cucurbit [8] uril (CB [8]) as a molecular host to encapsulate tris(2-phenylpyridine)iridium(III) (Ir(ppy)_3_), forming a stable Ir-CB [8] complex with enhanced electrochemiluminescent properties. The optimized AIECL system was subsequently integrated with a DNA walker-based biosensor for ultrasensitive miRNA-16 detection. Engineered DNA nanostructures triggered the directional displacement of quencher-modified AuNPs@NE from electrode surfaces through sequence-specific cleavage mechanisms. This spatial reorganization effectively restored the Ir-CB [8] ECL signal, enabling precise quantification at ultra-low concentrations. The synergistic combination of supramolecular engineering and dynamic DNA nanotechnology establishes a robust platform for clinical biomarker analysis with exceptional sensitivity and operational biocompatibility (Figure 11b).

#### 3.4.3. Protein Marker Analysis

Protein biomarkers serve as crucial biochemical indicators for monitoring physiological alterations across cellular, tissue, and systemic levels owing to their inherent molecular specificity [161,162]. The precise quantification of multiple biomarkers enables proactive disease prevention through early identification of pathological changes in biological systems.

**Figure 12:**
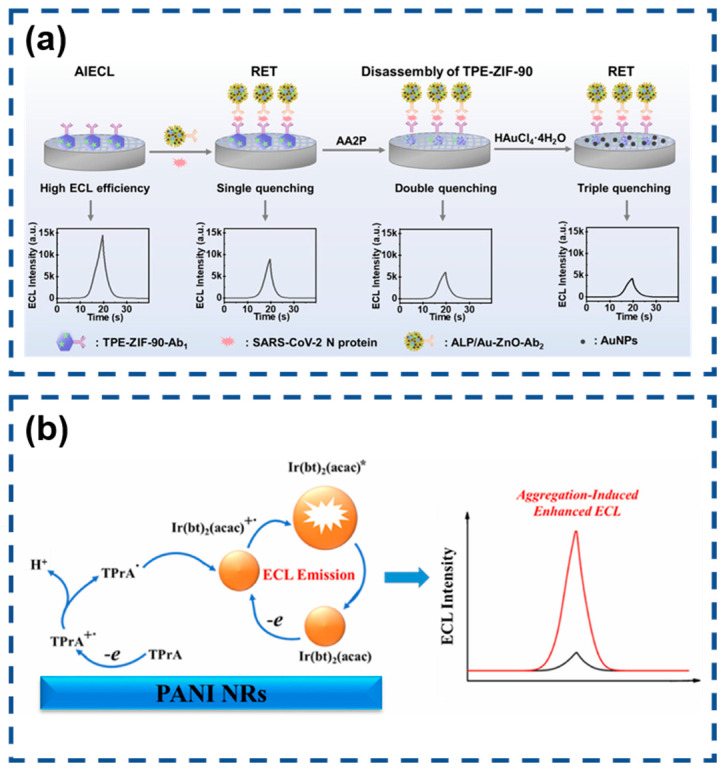
(**a**) Multiquenching-based SARS-CoV-2 N protein detector. Reproduced with permission from ref. [145]. Copyright 2024, American Chemical Society. (**b**) Sensitive electrochemiluminescence immunoassay of CD44 protein. (* indicates high-energy state molecules) Reproduced with permission from ref. [163]. Copyright 2025, Elsevier.

The global COVID-19 pandemic has intensified demands for advanced diagnostic technologies capable of sensitive SARS-CoV-2 detection. Wang’s team engineered an AIECL biosensor for the ultraprecise identification of SARS-CoV-2 nucleocapsid protein through innovative signal modulation strategies [145]. Their design employed ALP-functionalized gold nanoparticle-decorated ZnO nanoflowers (ALP/Au-ZnO) that enzymatically converted AA2P substrate into phosphate ions and ascorbic acid. This system achieved exceptional sensitivity through synergistic signal suppression mechanisms involving TPE-ZIF-90/Au-ZnO resonance energy transfer, zinc–phosphate coordination-induced structural disintegration, and in situ generated gold nanoparticle-mediated energy transfer. The cascaded quenching effects enabled remarkable detection performance with a 0.52 fg/mL limit of detection, demonstrating significant potential for early-stage viral infection diagnosis (Figure 12a).

Jia and co-workers developed an innovative encapsulation approach to enhance the performance of water-insoluble [Ir(bt)_2_(acac)] through nanoprecipitation with poly(styrene-maleic anhydride) (PSMA) [163]. This strategy effectively restricted the molecular motion of the iridium complex, inducing strong AIECL. The resulting Ir(bt)_2_(acac)-polymer dots (IrPdots) exhibited superior water dispersibility and significantly enhanced the ECL intensity compared to the free complex. The PSMA encapsulation introduced carboxyl functional groups absent in the native complex, enabling direct bioconjugation for biosensing applications. This advantage was exploited to construct a sandwich-type ECL immunosensor for CD44 detection, where IrPdots-labeled secondary antibodies served as ECL probes. The sensor platform utilized polyaniline nanorods (PANI NRs) to provide an enlarged electroactive surface for primary antibody immobilization. The optimized biosensor demonstrated excellent analytical performance, showing a logarithmic correlation between ECL intensity and CD44 concentration across a broad dynamic range (0.1 pg/mL to 50 ng/mL). The system achieved remarkable sensitivity with a 77 fg/mL detection limit, establishing its potential for clinical biomarker analysis (Figure 12b).

All the above cases illustrate the wide applications of AIECL illuminants in ECL biosensors, providing a theoretical basis for future market development. As illustrated in Table 1, typical electrochemiluminescent sensors employing aggregation-induced emission probes for biological detection are demonstrated.

## 4. Summary and Outlook

This review systematically examines the developmental trajectory, material design strategies, and application advancements of AIECL biosensors, highlighting their transformative potential for biosensors. We first delineate that, while many conventional ECL biosensors offer high sensitivity and near-zero background noise, certain luminophores suffer from ACQ, resulting in low emission efficiency in solid/aqueous phases and inadequate biocompatibility, thereby constraining clinical translation for these specific systems. The discovery of AIE provides a paradigm-shifting solution for overcoming ACQ in specific luminophores: AIEgens significantly suppress non-radiative decay via the restricted intramolecular motion (RIM) mechanism in aggregated states, achieving amplified luminescence efficiency. Building on this, Cola’s team pioneered the AIECL concept in 2017, demonstrating electrochemiluminescence enhancement in aggregated Pt(II) complexes through supramolecular self-assembly, establishing a novel ECL system with “aggregation-enhanced emission”, high biocompatibility, and environmental stability [83].

This work subsequently contrasts conventional and AIECL systems, focusing on AIE-guided luminophore design strategies encompassing metal clusters, small-molecule AIEgens, and polymeric AIE emitters. AIECL construction methodologies—including enzyme-triggered assembly, ligand–receptor interactions, supramolecular self-organization, and microencapsulation—optimize molecular packing and suppress non-radiative decay, substantially enhancing quantum yields and signal stability. Integrated with recognition elements (antibodies, aptamers, and peptides), AIECL platforms achieve ultrasensitive detection (aM–fg/mL level) of food contaminants (e.g., ciprofloxacin), environmental toxins, and disease biomarkers (e.g., dopamine, miRNA, SARS-CoV-2 nucleocapsid protein), demonstrating exceptional practical utility.

Despite significant progress, challenges impeding clinical translation and scalability persist: (1) Single-analyte limitation: Current AIE-based ECL sensors lack multiplexed detection capability for co-existing biomarkers in real samples, increasing costs and reducing accuracy. Additionally, reliance on single-signal quantification makes systems vulnerable to interference from background molecules in complex matrices (serum and cell lysates), which may disrupt aggregation or quench ECL signals. Developing ratiometric dual-signal ECL sensors using AIE materials could enhance robustness through signal ratio calibration. To overcome this constraint, developing multiplexed sensor arrays using orthogonal AIEgens and ratio metric signaling is proposed to enable concurrent multi-analyte detection. (2) Constrained ECL efficiency optimization: Current enhancement strategies depend on coreactant accelerators with inherent drawbacks—operational complexity, poor stability, and high cost. Intrinsic efficiency optimization via composition/structure/morphology engineering is urgently needed to minimize exogenous additives. Addressing this bottleneck requires pursuing intrinsic molecular engineering and nanomaterial hybridization to minimize exogenous additives. (3) Practical implementation barriers: Most AIECL biosensors remain laboratory prototypes. Complex designs (hierarchical assembly, rigid skeleton modification, and composite encapsulation) for stability enhancement substantially increase synthesis complexity and manufacturing costs, compromising operational simplicity, scalability, and cost-effectiveness essential for clinical/industrial deployment. For scalable deployment, adopting high-throughput manufacturing and integrated systems emerges as a viable pathway to streamline production and operation.

In summary, AIECL biosensors provide revolutionary tools for life science analysis. Future research must advance mechanistic understanding, biosafety assessment, and technology integration to propel this field toward high-precision, multifunctional, clinically viable platforms for early disease diagnosis and personalized medicine.

## Figures and Tables

**Table 1:** Representative AIECL biosensors in bioanalytical detection.

Target Analyte	Sensing Strategies	AIECL Emitters	Linear Range	LOD	Reference
ALP	Turn off	Ir(ppy)3@apoFt bioconjugate (TPrA)	0.1–6.0 U L^−1^	0.037 U L^−1^	[164]
GSH	Turn off	PS-BZIMPY-Pt	0.1–200 μM	0.016 μM	[146]
CYFRA 21-1	Turn on	Pdots (TPrA)	1 pg mL^−1^ to 50 ng mL^−1^	0.43 pg mL^−1^	[165]
Cr(vi)	Turn on	TPBS-C (S_2_O_8_^2−^)	10^−12^ to 10^−4^ M	0.83 pM	[166]
Chloramphenicol	Turn on	BDT-TPA (TEOA)	5 × 10^−13^ to 4 × 10^−10^ M	1.18 × 10^−13^ M	[167]
TI	Turn on	MOG (S_2_O_8_^2−^)	0.1 pM to 10 nM	20 fM	[168]
NSE	Turn off	TP-CCOH NCs (S_2_O_8_^2−^)	0.0001 to 10 ng mL^−1^	52 fg mL^−1^	[169]
cTnI	Turn off	TCPP J-aggregate (S_2_O_8_^2−^)	0-100 µg L^−1^	43 fg mL^−1^	[170]
Cu^2+^	ECL-RET	TPE-(NO_2_)_4_ (S_2_O_8_^2−^)	1.0 pM to 500 nM	0.33 pM	[45]
MUC1	Turn on	Zr-TCBPE-PEI	1 fg mL^−1^ to 1 ng mL^−1^	0.29 fg mL^−1^	[139]
CYFRA 21-1	Turn off	CIPNPs (TPA)	10^−7^ to 500 ng mL^−1^	0.01471 pg mL^−1^	[171]
PiRNA-823	Turn on	Zn-MOG	100 aM to 100 pM	60.0 aM	[172]
NO	Turn off	[Ru(phen)_2_(phen–Br_2_)]^2+^	2 nM to 2 × 10^5^ nM	2 nM	[173]
Aflatoxins B1 (AFB1)	Turn on	TH-UiO-66-NH_2_	10 fg mL^−1^ to 1 μg mL^−1^	1.04 fg mL^−1^	[174]
Malathion	Turn on	poly [2,5-dioctyl-1,4-phenylene] (PDP)	5.0 fM to 0.5 μM	0.9 fM	[149]
Dopamine (DA) and H_2_O_2_	Turn off	[Ir(pbi)_2_(acac)]	0.01–100 nmol/mL	2.0 pmol/mL	[151]
Deoxynivalenol (DON)	Turn on	piperazine-functionalized carbon dot aggregates (P-CDAs)	1 fg/mL to 10 ng/mL	0.112 fg/mL	[175]
Zearalenone (ZEN)	Turn on	TPE NAs	1 fg/mL–100 ng/mL	0.362 fg/mL	[176]
Chloramphenicol (CAP)	Turn on	1,1,2,2-tetrakis(4-(pyridin-4-yl) phenyl)-ethene (TPPE)	10 fmol·L^−1^ to 100nmol·L^−1^	1.81 fmol·L^−1^	[177]
Tetracycline	Turn on	tetrakis(4-aminophenyl)ethene (ETTA)	0.1 pM∼1 μM	42.6 fM	[178]
microRNA-21	Turn on	AuNPs@MXene	100 aM to 11nM	31 aM	[179]
miRNA-141	Turn on	Al–H_3_NTB-MOG	10 aM to 1 nM	6.48 aM	[180]

## Data Availability

Not applicable.

## References

[1] Fenn J.B., Mann M., Meng C.K., Wong S.F., Whitehouse C.M. (1989). Electrospray Ionization for Mass Spectrometry of Large Biomolecules. Science.

[2] Bao G., Suresh S. (2003). Cell and molecular mechanics of biological materials. Nat. Mater..

[3] Nel A.E., Mädler L., Velegol D., Xia T., Hoek E.M.V., Somasundaran P., Klaessig F., Castranova V., Thompson M. (2009). Understanding biophysicochemical interactions at the nano–bio interface. Nat. Mater..

[4] Zhuang J., Young A.P., Tsung C.-K. (2017). Integration of Biomolecules with Metal–Organic Frameworks. Small.

[5] An H., Li M., Gao J., Zhang Z., Ma S., Chen Y. (2019). Incorporation of biomolecules in Metal-Organic Frameworks for advanced applications. Coordin. Chem. Rev..

[6] Mu J., He L., Huang P., Chen X. (2019). Engineering of nanoscale coordination polymers with biomolecules for advanced applications. Coordin. Chem. Rev..

[7] Thome C.P., Hoertdoerfer W.S., Bendorf J.R., Lee J.G., Shields C.W.I.V. (2023). Electrokinetic Active Particles for Motion-Based Biomolecule Detection. Nano Lett..

[8] Riviere-Cazaux C., Keough M.B., Zuccato J.A., Kumar R., Schulz S., Warrington A.E., Ruff M.W., Ellingson B.M., Sanai N., Campian J.L. (2025). A hitchhiker’s guide to cerebrospinal fluid biomarkers for neuro-oncology. Neuro-Oncology.

[9] Medintz I.L., Uyeda H.T., Goldman E.R., Mattoussi H. (2005). Quantum dot bioconjugates for imaging, labelling and sensing. Nat. Mater..

[10] Zhou J., Liu Z., Li F. (2012). Upconversion nanophosphors for small-animal imaging. Chem. Soc. Rev..

[11] Zhang P., Cui Y., Anderson C.F., Zhang C., Li Y., Wang R., Cui H. (2018). Peptide-based nanoprobes for molecular imaging and disease diagnostics. Chem. Soc. Rev..

[12] Kwon T., Gunasekaran S., Eom K. (2019). Atomic force microscopy-based cancer diagnosis by detecting cancer-specific biomolecules and cells. BBA-REV Cancer.

[13] Hu X.-L., Kwon N., Yan K.-C., Sedgwick A.C., Chen G.-R., He X.-P., James T.D., Yoon J. (2020). Bio-Conjugated Advanced Materials for Targeted Disease Theranostics. Adv. Funct. Mater..

[14] Chen W., Li J., Guo J., Li L., Wu H. (2024). Diagnosis and therapy of Alzheimer’s disease: Light-driven heterogeneous redox processes. Adv. Collioid. Interface Sci..

[15] Saini N., Kriti, Thakur A., Saini S., Kaur N., Singh N. (2025). Synergizing Machine Learning and fluorescent biomolecules: A new era in sensing platforms. Trac-Trend. Anal. Chem..

[16] Woo H.-K., Nam Y., Park H.G., Lee H. (2025). Bridging laboratory innovation to translational research and commercialization of extracellular vesicle isolation and detection. Biosens. Bioelectron..

[17] Anker J.N., Hall W.P., Lyandres O., Shah N.C., Zhao J., Van Duyne R.P. (2008). Biosensing with plasmonic nanosensors. Nat. Mater..

[18] Saha K., Agasti S.S., Kim C., Li X., Rotello V.M. (2012). Gold Nanoparticles in Chemical and Biological Sensing. Chem. Rev..

[19] Maduraiveeran G., Sasidharan M., Ganesan V. (2018). Electrochemical sensor and biosensor platforms based on advanced nanomaterials for biological and biomedical applications. Biosens. Bioelectron..

[20] Zhao Y., Tong R.-J., Xia F., Peng Y. (2019). Current status of optical fiber biosensor based on surface plasmon resonance. Biosens. Bioelectron..

[21] Wang L., Lin J. (2020). Recent advances on magnetic nanobead based biosensors: From separation to detection. Trac-Trend. Anal. Chem..

[22] Tang R., Yang J., Shao C., Shen N., Chen B., Gu Y., Li C., Xu D., Guo C. (2025). Two-dimensional nanomaterials-based optical biosensors empowered by machine learning for intelligent diagnosis. Trac-Trend. Anal. Chem..

[23] Zhu Y., Cheng Z., Wang X., Zhang C., Li X., Wei Y., Wang J., Fang Y., Wang Y., Zhang D. (2025). Synergistic optimization strategies for the development of multienzymatic cascade system-based electrochemical biosensors with enhanced performance. Biosens. Bioelectron..

[24] Vacher M., Fdez. Galván I., Ding B.-W., Schramm S., Berraud-Pache R., Naumov P., Ferré N., Liu Y.-J., Navizet I., Roca-Sanjuán D. (2018). Chemi- and Bioluminescence of Cyclic Peroxides. Chem. Rev..

[25] Richter M.M. (2004). Electrochemiluminescence (ECL). Chem. Rev..

[26] Miao W. (2008). Electrogenerated Chemiluminescence and Its Biorelated Applications. Chem. Rev..

[27] Chen D., Tang L., Li J. (2010). Graphene-based materials in electrochemistry. Chem. Soc. Rev..

[28] Fosdick S.E., Knust K.N., Scida K., Crooks R.M. (2013). Bipolar Electrochemistry. Angew. Chem. Int. Edit..

[29] Knežević S., Han D., Liu B., Jiang D., Sojic N. (2024). Electrochemiluminescence Microscopy. Angew. Chem. Int. Edit..

[30] Li Y.-X., Dai Y.-X., Chauvin J., Zhang X.-J., Cosnier S., Shan D. (2024). Intermolecular forces and assembly strategies in porphyrin-based electrochemiluminescence: Mechanisms and future prospects. Trac-Trend. Anal. Chem..

[31] Zhang Y., Xu Y., Li J., Chen R., Chen W., Peng H. (2024). Essential role of electrocatalysis in electrochemiluminescence: Recent advances and perspectives. Trac-Trend. Anal. Chem..

[32] Bertoncello P., Forster R.J. (2009). Nanostructured materials for electrochemiluminescence (ECL)-based detection methods: Recent advances and future perspectives. Biosens. Bioelectron..

[33] Hu L., Xu G. (2010). Applications and trends in electrochemiluminescence. Chem. Soc. Rev..

[34] Zhang L., Wang E. (2014). Metal nanoclusters: New fluorescent probes for sensors and bioimaging. Nano Today.

[35] Bansod B., Kumar T., Thakur R., Rana S., Singh I. (2017). A review on various electrochemical techniques for heavy metal ions detection with different sensing platforms. Biosens. Bioelectron..

[36] Farka Z., Juřík T., Kovář D., Trnková L., Skládal P. (2017). Nanoparticle-Based Immunochemical Biosensors and Assays: Recent Advances and Challenges. Chem. Rev..

[37] Khalil M., Teunissen C.E., Otto M., Piehl F., Sormani M.P., Gattringer T., Barro C., Kappos L., Comabella M., Fazekas F. (2018). Neurofilaments as biomarkers in neurological disorders. Nat. Rev. Neurol..

[38] Ma C., Cao Y., Gou X., Zhu J.-J. (2020). Recent Progress in Electrochemiluminescence Sensing and Imaging. Anal. Chem..

[39] Roda A., Mirasoli M., Michelini E., Di Fusco M., Zangheri M., Cevenini L., Roda B., Simoni P. (2016). Progress in chemical luminescence-based biosensors: A critical review. Biosens. Bioelectron..

[40] Chen Y., Zhou S., Li L., Zhu J.-J. (2017). Nanomaterials-based sensitive electrochemiluminescence biosensing. Nano Today.

[41] Jin H., Gui R., Yu J., Lv W., Wang Z. (2017). Fabrication strategies, sensing modes and analytical applications of ratiometric electrochemical biosensors. Biosens. Bioelectron..

[42] Babamiri B., Bahari D., Salimi A. (2019). Highly sensitive bioaffinity electrochemiluminescence sensors: Recent advances and future directions. Biosens. Bioelectron..

[43] Han Q., Wang H., Wang J. (2024). Multi-Mode/Signal Biosensors: Electrochemical Integrated Sensing Techniques. Adv. Funct. Mater..

[44] Song K., Zhao W., Zhou Y., Liu D., Chu P.K. (2024). Innovative strategies in metal-organic frameworks for enhanced electrochemiluminescence biosensors. Coordin. Chem. Rev..

[45] Han Q., Wang C., Li Z., Wu J., Liu P.K., Mo F., Fu Y. (2020). Multifunctional Zinc Oxide Promotes Electrochemiluminescence of Porphyrin Aggregates for Ultrasensitive Detection of Copper Ion. Anal. Chem..

[46] Hua Q., Tang F., Wang X., Li M., Gu X., Sun W., Luan F., Tian C., Zhuang X. (2022). Electrochemiluminescence sensor based on EuS nanocrystals for ultrasensitive detection of mercury ions in seafood. Sensor Actuat. B-Chem..

[47] Kotopoulou S., Zampelas A., Magriplis E. (2022). Dietary nitrate and nitrite and human health: A narrative review by intake source. Nutr. Rev..

[48] Li Y., Zhou H., Zhang J., Cui B., Fang Y. (2022). Determination of nitrite in food based on its sensitizing effect on cathodic electrochemiluminescence of conductive PTH-DPP films. Food Chem..

[49] Zhao M., Chen A.-Y., Huang D., Zhuo Y., Chai Y.-Q., Yuan R. (2016). Cu Nanoclusters: Novel Electrochemiluminescence Emitters for Bioanalysis. Anal. Chem..

[50] He Y., Hu F., Zhao J., Yang G., Zhang Y., Chen S., Yuan R. (2021). Bifunctional Moderator-Powered Ratiometric Electrochemiluminescence Enzymatic Biosensors for Detecting Organophosphorus Pesticides Based on Dual-Signal Combined Nanoprobes. Anal. Chem..

[51] Li J., Wang X., Liu W., Li X., Yang L., Ma H., Wu R., Wei Q. (2021). Highly selective electrochemiluminescence aptasensor coupled with mesoporous Fe_3_O_4_@Cu@Cu_2_O as co-reaction accelerator for ATP assay based on target-triggered emitter release. Sensor Actuat. B-Chem..

[52] Sun Y., Li P., Zhu Y., Zhu X., Zhang Y., Liu M., Liu Y. (2021). In situ growth of TiO2 nanowires on Ti_3_C_2_ MXenes nanosheets as highly sensitive luminol electrochemiluminescent nanoplatform for glucose detection in fruits, sweat and serum samples. Biosens. Bioelectron..

[53] Zhang M., Qian M., Huang H., Gao Q., Zhang C., Qi H. (2022). Carboxyl group bearing iridium(III) solvent complex as photoluminescence and electrochemiluminescence probe for the detection of histidine. ElectroAnal. Chem..

[54] Kinoshita E., Kinoshita-Kikuta E., Takiyama K., Koike T. (2006). Phosphate-binding Tag, a New Tool to Visualize Phosphorylated Proteins *. Mol. Cell. Proteomics.

[55] Luo Q.-X., Li Y., Liang R.-P., Cao S.-P., Jin H.-J., Qiu J.-D. (2020). Gold nanoclusters enhanced electrochemiluminescence of g-C3N4 for protein kinase activity analysis and inhibition. ElectroAnal. Chem..

[56] Li B., Huang X., Lu Y., Fan Z., Li B., Jiang D., Sojic N., Liu B. (2022). High Electrochemiluminescence from Ru(bpy)32+ Embedded Metal–Organic Frameworks to Visualize Single Molecule Movement at the Cellular Membrane. Adv. Sci..

[57] Wang C., Liu S., Ju H. (2023). Electrochemiluminescence nanoemitters for immunoassay of protein biomarkers. Bioelectrochemistry.

[58] Croce C.M. (2009). Causes and consequences of microRNA dysregulation in cancer. Nat. Rev. Genet..

[59] Lin S., Gregory R.I. (2015). MicroRNA biogenesis pathways in cancer. Nat. Rev. Cancer.

[60] Liu H., Zhou X., Liu W., Yang X., Xing D. (2016). Paper-Based Bipolar Electrode Electrochemiluminescence Switch for Label-Free and Sensitive Genetic Detection of Pathogenic Bacteria. Anal. Chem..

[61] Liu J.-L., Tang Z.-L., Zhang J.-Q., Chai Y.-Q., Zhuo Y., Yuan R. (2018). Morphology-Controlled 9,10-Diphenylanthracene Nanoblocks as Electrochemiluminescence Emitters for MicroRNA Detection with One-Step DNA Walker Amplification. Anal. Chem..

[62] Liu P.-F., Zhao K.-R., Liu Z.-J., Wang L., Ye S.-Y., Liang G.-X. (2021). Cas12a-based electrochemiluminescence biosensor for target amplification-free DNA detection. Biosens. Bioelectron..

[63] Ning Z., Chen M., Wu G., Zhang Y., Shen Y. (2021). Recent advances of functional nucleic acids-based electrochemiluminescent sensing. Biosens. Bioelectron..

[64] Wang Y., Fei Y., Yang T., Luo Z., Xu Y., Su B., Lin X. (2023). Nanotechnology for ultrafast nucleic acid amplification. Nano Today.

[65] Dolci L.S., Zanarini S., Ciana L.D., Paolucci F., Roda A. (2009). Development of a New Device for Ultrasensitive Electrochemiluminescence Microscopy Imaging. Anal. Chem..

[66] Zhou J., Ma G., Chen Y., Fang D., Jiang D., Chen H.-Y. (2015). Electrochemiluminescence Imaging for Parallel Single-Cell Analysis of Active Membrane Cholesterol. Anal. Chem..

[67] Liu G., Ma C., Jin B.-K., Chen Z., Zhu J.-J. (2018). Direct Electrochemiluminescence Imaging of a Single Cell on a Chitosan Film Modified Electrode. Anal. Chem..

[68] Voci S., Goudeau B., Valenti G., Lesch A., Jović M., Rapino S., Paolucci F., Arbault S., Sojic N. (2018). Surface-Confined Electrochemiluminescence Microscopy of Cell Membranes. J. Am. Chem. Soc..

[69] Ding H., Guo W., Su B. (2020). Imaging Cell-Matrix Adhesions and Collective Migration of Living Cells by Electrochemiluminescence Microscopy. Angew. Chem. Int. Edit..

[70] Ding H., Zhou P., Fu W., Ding L., Guo W., Su B. (2021). Spatially Selective Imaging of Cell–Matrix and Cell–Cell Junctions by Electrochemiluminescence. Angew. Chem. Int. Edit..

[71] Liu Y., Zhang H., Li B., Liu J., Jiang D., Liu B., Sojic N. (2021). Single Biomolecule Imaging by Electrochemiluminescence. J. Am. Chem. Soc..

[72] Ding L., Ding H., Zhou P., Xi L., Su B. (2022). Surface-Sensitive Imaging Analysis of Cell–Microenvironment Interactions by Electrochemiluminescence Microscopy. Anal. Chem..

[73] Descamps J., Colin C., Tessier G., Arbault S., Sojic N. (2023). Ultrasensitive Imaging of Cells and Sub-Cellular Entities by Electrochemiluminescence. Angew. Chem. Int. Edit..

[74] Dong J., Feng J. (2023). Electrochemiluminescence from Single Molecule to Imaging. Anal. Chem..

[75] Gou X., Xing Z., Ma C., Zhu J.-J. (2023). A Close Look at Mechanism, Application, and Opportunities of Electrochemiluminescence Microscopy. CBMI.

[76] Lu Y., Huang X., Wang S., Li B., Liu B. (2023). Nanoconfinement-Enhanced Electrochemiluminescence for in Situ Imaging of Single Biomolecules. ACS Nano.

[77] Zhou P., Ding L., Yan Y., Wang Y., Su B. (2023). Recent advances in label-free imaging of cell–matrix adhesions. Chem. Commun..

[78] Zhu W., Dong J., Ruan G., Zhou Y., Feng J. (2023). Quantitative Single-Molecule Electrochemiluminescence Bioassay. Angew. Chem. Int. Edit..

[79] Chen Y., Min X., Zhang X., Zhang F., Lu S., Xu L.-P., Lou X., Xia F., Zhang X., Wang S. (2018). AIE-based superwettable microchips for evaporation and aggregation induced fluorescence enhancement biosensing. Biosens. Bioelectron..

[80] Huang Y., Wang Z., Chen Z., Zhang Q. (2019). Organic Cocrystals: Beyond Electrical Conductivities and Field-Effect Transistors (FETs). Angew. Chem. Int. Edit..

[81] Habenicht S.H., Kupfer S., Nowotny J., Schramm S., Weiß D., Beckert R., Görls H. (2018). Highly fluorescent single crystals of a 4-ethoxy-1,3-thiazole. Dyes Pigments.

[82] Luo J., Xie Z., Lam J.W.Y., Cheng L., Chen H., Qiu C., Kwok H.S., Zhan X., Liu Y., Zhu D. (2001). Aggregation-induced emission of 1-methyl-1,2,3,4,5-pentaphenylsilole. Chem. Commun..

[83] Carrara S., Aliprandi A., Hogan C.F., De Cola L. (2017). Aggregation-Induced Electrochemiluminescence of Platinum(II) Complexes. J. Am. Chem. Soc..

[84] Wei X., Zhu M.-J., Yan H., Lu C., Xu J.-J. (2019). Recent Advances in Aggregation-Induced Electrochemiluminescence. Chem. Eur. J..

[85] Lv X., Li Y., Cui B., Fang Y., Wang L. (2022). Electrochemiluminescent sensor based on an aggregation-induced emission probe for bioanalytical detection. Analyst.

[86] Moreno-Alcántar G., Aliprandi A., De Cola L., Tang Y., Tang B.Z. (2022). Aggregation-Induced Emission in Electrochemiluminescence: Advances and Perspectives. Aggregation-Induced Emission.

[87] Dong Z., Du F., Zhang W., Tian Y., Xu G. (2025). Recent advances in Tetraphenylethylene-based aggregation-induced electrochemiluminescence for biosensing applications. Curr. Opin. Electrochem..

[88] Deng M.-Z., Zhong M.-Y., Li M.-L., Huang G.-Q., He H., Xiao X., Bai R.-B., Ukwatta R.H., Mi L., Zhang T.-T. (2025). Research progress on electrochemiluminescence nanomaterials and their applications in biosensors—A review. Anal. Chim. Acta.

[89] Wang X., Wan R., Tang Y., Sun S., Chen H., Li L., Chen J., Wei J., Chi Z., Li H. (2025). Aggregation-induced emission materials-based Electrochemiluminescence emitters for sensing applications: Progress, challenges and perspectives. Coordin. Chem. Rev..

[90] Zhang D., Li C., Xiang T., Yu Y., Xu R., Zhang Y. (2025). Classification and research progress of aggregation-induced electrochemiluminescence materials for sensoring application. Microchem. J..

[91] Dufford R.T., Nightingale D., Gaddum L.W. (1927). Luminescence of Grignard Compounds in Electric and Magnetic Fields, and Related Electrical Phenomena. J. Am. Chem. Soc..

[92] Harvey N. (1929). Luminescence during Electrolysis. J. Phys. Chem. C.

[93] Hercules D.M. (1964). Chemiluminescence Resulting from Electrochemically Generated Species. Science.

[94] Tokel N.E., Bard A.J. (1972). Electrogenerated chemiluminescence. IX. Electrochemistry and emission from systems containing tris(2,2′-bipyridine)ruthenium(II) dichloride. J. Am. Chem. Soc..

[95] Noffsinger J.B., Danielson N.D. (1987). Generation of chemiluminescence upon reaction of aliphatic amines with tris(2,2′-bipyridine)ruthenium(III). Anal. Chem..

[96] Schramm S., Karothu D.P., Lui N.M., Commins P., Ahmed E., Catalano L., Li L., Weston J., Moriwaki T., Solntsev K.M. (2019). Thermochemiluminescent peroxide crystals. Nat. Commun..

[97] Moroni G., Calabria D., Quintavalla A., Lombardo M., Mirasoli M., Roda A., Gioiello A. (2022). Thermochemiluminescence-Based Sensitive Probes: Synthesis and Photophysical Characterization of Acridine-Containing 1,2-Dioxetanes Focusing on Fluorophore Push-Pull Effects. ChemPhotoChem.

[98] Fleet B., Keliher P.N., Kirkbright G.F., Pickford C.J. (1969). Some observations on the analytical usefulness of electrochemiluminescence for the determination of microgram amounts of aromatic hydrocarbons. Analyst.

[99] Knight A.W., Greenway G.M. (1995). Electrogenerated chemiluminescent determination of pyruvate using tris(2,2′-bipyridine)ruthenium(II). Analyst.

[100] Gross E.M., Anderson J.D., Slaterbeck A.F., Thayumanavan S., Barlow S., Zhang Y., Marder S.R., Hall H.K., Nabor M.F., Wang J.F. (2000). Electrogenerated Chemiluminescence from Derivatives of Aluminum Quinolate and Quinacridones:  Cross-Reactions with Triarylamines Lead to Singlet Emission through Triplet-Triplet Annihilation Pathways. J. Am. Chem. Soc..

[101] Kerr E., Doeven E.H., Barbante G.J., Hogan C.F., Bower D.J., Donnelly P.S., Connell T.U., Francis P.S. (2015). Annihilation electrogenerated chemiluminescence of mixed metal chelates in solution: Modulating emission colour by manipulating the energetics. Chem. Sci..

[102] Wu F., Feng Y., Chi Y. (2016). Yellow electrochemiluminescence emission from hydrophilic poly[(9,9-di-(2-ethylhexyl)-9H-fluorene-2,7-vinylene)-co-(1-methoxy-4-(2-ethylhe-xyloxy)-2,5-phenylenevinylene)] (PFV) conjugated polymer dots capped with Triton X-100 in aqueous solution. ElectroAnal. Chem..

[103] Chu K., Adsetts J.R., He S., Zhan Z., Yang L., Wong J.M., Love D.A., Ding Z. (2020). Electrogenerated Chemiluminescence and Electroluminescence of N-Doped Graphene Quantum Dots Fabricated from an Electrochemical Exfoliation Process in Nitrogen-Containing Electrolytes. Chem. Eur. J..

[104] Li T., Su Y., Zhang L., Song H., Lv Y. (2025). The development of electrochemiluminescent probes: Mechanism and application. Mircochem. J..

[105] Sobhanie E., Salehnia F., Xu G., Hamidipanah Y., Arshian S., Firoozbakhtian A., Hosseini M., Ganjali M.R., Hanif S. (2022). Recent trends and advancements in electrochemiluminescence biosensors for human virus detection. Trac-Trend. Anal. Chem..

[106] Factor B., Muegge B., Workman S., Bolton E., Bos J., Richter M.M. (2001). Surfactant Chain Length Effects on the Light Emission of Tris(2,2‘-bipyridyl)ruthenium(II)/Tripropylamine Electrogenerated Chemiluminescence. Anal. Chem..

[107] Kuwabara T., Noda T., Ohtake H., Ohtake T., Toyama S., Ikariyama Y. (2003). Classification of DNA-binding mode of antitumor and antiviral agents by the electrochemiluminescence of ruthenium complex. Anal. Biochem..

[108] Herbert M.B., Marx V.M., Pederson R.L., Grubbs R.H. (2013). Concise Syntheses of Insect Pheromones Using Z-Selective Cross Metathesis. Angew. Chem. Int. Edit..

[109] Li X., Huang Y., Chen J., Zhuo S., Lin Z., Chen J. (2022). A highly sensitive homogeneous electrochemiluminescence biosensor for flap endonuclease 1 based on branched hybridization chain reaction amplification and ultrafiltration separation. Bioelectrochemistry.

[110] Zhang Y., Yan X., Liu D., Jie G. (2022). Versatile electrochemiluminescence sensor for dual-potential “off” and “on” detection of double targets based on a novel terbium organic gel and multifunctional DNA network probes. Sensor Actuat. B-Chem..

[111] Peng Y., Wang Z.-G., Qi B.-P., Liu C., Tang B., Zhang Z.-L., Liu S.-L., Pang D.-W. (2024). Carboxyl groups on carbon nanodots as co-reactant sites for anodic electrochemiluminescence of tris(2,2-bipyridine)ruthenium(II). J. Colloid. Interface Sci..

[112] Adamson N.S., Blom S.J., Doeven E.H., Connell T.U., Hadden C., Knežević S., Sojic N., Fracassa A., Valenti G., Paolucci F. (2024). Electrochemiluminescence Enhanced by a Non-Emissive Dual Redox Mediator. Angew. Chem. Int. Edit..

[113] Fernández-Hernández J.M., Ladouceur S., Shen Y., Iordache A., Wang X., Donato L., Gallagher-Duval S., de Anda Villa M., Slinker J.D., De Cola L. (2013). Blue light emitting electrochemical cells incorporating triazole-based luminophores. J. Mater. Chem..

[114] Barbante G.J., Doeven E.H., Kerr E., Connell T.U., Donnelly P.S., White J.M., Lópes T., Laird S., Wilson D.J.D., Barnard P.J. (2014). Understanding Electrogenerated Chemiluminescence Efficiency in Blue-Shifted Iridium(III)-Complexes: An Experimental and Theoretical Study. Chem. Eur. J..

[115] Song L., Kuang G., Zhang G., Guo J., Fu Y. (2023). New luminescent hydrophilic iridium(III) nanoflower at low potential for electrochemiluminescence immunosensing. Chem. Eng. J..

[116] Dai C., Mao Z., Xu Y., Jia J., Tang H., Zhao Y., Zhou Y. (2024). Bis-tridentate Iridium(III) Complex with the N-Heterocyclic Carbene Ligand as a Novel Efficient Electrochemiluminescence Emitter for the Sandwich Immunoassay of the HHV-6A Virus. Anal. Chem..

[117] Dai C., Xu Y., Ke L., Zhu M., Deng R., Wang X., Zhou Y. (2025). Multiple-Signal Amplification Strategy to Fabricate an Ultrasensitive Electrochemiluminescence Magnetic Immunosensor for Detecting Biomarkers of Alzheimer’s Disease via Iridium-Based Self-Enhancing Nanoemitters. ACS Sens..

[118] Xiuhua W., Chao L., Yifeng T. (2012). Microemulsion-enhanced electrochemiluminescence of luminol-H2O2 for sensitive flow injection analysis of antioxidant compounds. Talanta.

[119] Fereja T.H., Wang C., Liu F., Guan Y., Xu G. (2020). A high-efficiency cathodic sodium nitroprusside/luminol/H2O2 electrochemiluminescence system in neutral media for the detection of sodium nitroprusside, glucose, and glucose oxidase. Analyst.

[120] Wu L., Ding F., Yin W., Ma J., Wang B., Nie A., Han H. (2017). From Electrochemistry to Electroluminescence: Development and Application in a Ratiometric Aptasensor for Aflatoxin B1. Anal. Chem..

[121] Lu H.-J., Zhao W., Xu J.-J., Chen H.-Y. (2018). Visual electrochemiluminescence ratiometry on bipolar electrode for bioanalysis. Biosens. Bioelectron..

[122] Yang L., Jia Y., Wu D., Zhang Y., Ju H., Du Y., Ma H., Wei Q. (2019). Synthesis and Application of CeO2/SnS2 Heterostructures as a Highly Efficient Coreaction Accelerator in the Luminol–Dissolved O2 System for Ultrasensitive Biomarkers Immunoassay. Anal. Chem..

[123] Li J.-H., Liu J.-L., Zhang X.-L., Zhu X.-C., Yuan R., Chai Y.-Q. (2023). Ultrasensitive Electrochemiluminescence Biosensor Based on 2D Co3O4 Nanosheets as a Coreaction Accelerator and Highly Ordered Rolling DNA Nanomachine as a Signal Amplifier for the Detection of MicroRNA. Anal. Chem..

[124] Li J., Yang J., Zhu L., Liu Y., He Y., Li Y. (2024). Ultrastable Luminol–OH–-(Ni-WOx-CNT) ECL System with High Strength and Its Applications in Sensing. Anal. Chem..

[125] Zhang X., Wang X., Zhu L., Zhu J., Zheng Q., Yuan J., Xu W., Cao J. (2025). Target responsive-regulated CRISPR/Cas12a electrochemiluminescence sensing of salmonella typhimurium integrating ultrafine Pt NCs-anchored MXenes-boosted luminol/O2 system. Biosens. Bioelectron..

[126] Ding Z., Quinn B.M., Haram S.K., Pell L.E., Korgel B.A., Bard A.J. (2002). Electrochemistry and Electrogenerated Chemiluminescence from Silicon Nanocrystal Quantum Dots. Science.

[127] Zhu R., Ding Z. (2005). Enhancing image quality of scanning electrochemical microscopy by improved probe fabrication and displacement. Can. J. Chem..

[128] Lu C., Zhou J., Lipson R.H., Ding Z. (2009). Simple method to fabricate large scale quantum dot architectures. Mater. Lett..

[129] Zhou J., Liu H., Wang F., Simpson T., Sham T.-K., Sun X., Ding Z. (2013). An electrochemical approach to fabricating honeycomb assemblies from multiwall carbon nanotubes. Carbon.

[130] Sun H., Cao Z., Qin H., Peng X., Su B. (2024). Ligand-controlled electrochemiluminescence generation from CdSe/CdS/ZnS core/shell/shell quantum dots. Nano Res..

[131] Gao X., Tian Z., Ren X., Ai Y., Zhang B., Zou G. (2024). Silver Nanocluster-Tagged Electrochemiluminescence Immunoassay with a Sole and Narrow Triggering Potential Window. Anal. Chem..

[132] Liu L.-L., Xiang L., Chai Y.-Q., Yuan R. (2024). Confinement-enhanced electrochemiluminescence of copper nanoclusters on 3D layered double hydroxide for ultrasensitive detection of GFAP. Biosens. Bioelectron..

[133] Zhao Z., He B., Tang B. (2015). Aggregation-induced emission of siloles. Chem. Sci..

[134] Wang H., Li Q., Alam P., Bai H., Bhalla V., Bryce M.R., Cao M., Chen C., Chen S., Chen X. (2023). Aggregation-Induced Emission (AIE), Life and Health. ACS Nano.

[135] Wang D., Nie Y., Li Z., Ma Q. (2023). The controllable assembly of Cu nanocluster-based aggregation induced ECL strategy for miRNA detection. Anal. Chim. Acta.

[136] Wang D., Nie Y., Wang P., Ma Q. (2023). In situ synthesis of Cu nanoclusters/CeO2 nanorod as aggregated induced ECL probe for triple-negative breast cancer detection. Talanta.

[137] Hesari M., Workentin M.S., Ding Z. (2014). Highly efficient electrogenerated chemiluminescence of Au_38_ Nanoclusters. ACS Nano.

[138] Shen Z., Yang Y., Guo Y., Chai Y., Liu J., Ruo Y. (2023). Zn^2+^-induced gold cluster aggregation enhanced electrochemiluminescence for ultrasensitive detection of microRNA-21. Anal. Chem..

[139] Jiang M.-H., Li S.-K., Zhong X., Liang W.-B., Chai Y.-Q., Zhuo Y., Yuan R. (2019). Electrochemiluminescence Enhanced by Restriction of Intramolecular Motions (RIM): Tetraphenylethylene Microcrystals as a Novel Emitter for Mucin 1 Detection. Anal. Chem..

[140] Huang W., Hu G.-B., Yao L.-Y., Yang Y., Liang W.-B., Yuan R., Xiao D.-R. (2020). Matrix Coordination-Induced Electrochemiluminescence Enhancement of Tetraphenylethylene-Based Hafnium Metal–Organic Framework: An Electrochemiluminescence Chromophore for Ultrasensitive Electrochemiluminescence Sensor Construction. Anal. Chem..

[141] Zheng G., Hu S., Qin D., Nong C., Yang L., Deng B. (2023). Aggregation-induced electrochemiluminescence enhancement of Ag-MOG for amyloid β 42 sensing. Anal. Chim. Acta.

[142] Wang J., Yao L., Huang W., Yang Y., Liang W., Yuan R., Xiao D. (2021). Overcoming aggregation-induced quenching by metal-organic framework for electrochemiluminescence (ECL) enhancement: Zn-PTC as a new ECL emitter for ultrasensitive micrornas detection. ACS Appl. Mater. Interfaces.

[143] Ren X., Zhang D., Li C., Zhao J., Feng R., Zhang Y., Xu R., Wei Q. (2024). Europium metal-organic framework with a tetraphenylethylene-based ligand: A dual-mechanism quenching immunosensor for enhanced electrochemiluminescence via the coordination trigger. Anal. Chem..

[144] Fu L., Gao X., Dong S., Jia J., Xu Y., Wang D., Zou G. (2022). Coreactant-Free and Direct Electrochemiluminescence from Dual-Stabilizer-Capped InP/ZnS Nanocrystals: A New Route Involving n-Type Luminophore. Anal. Chem..

[145] Wang W., Kan X. (2024). Multiquenching-Based Aggregation-Induced Electrochemiluminescence Sensing for Highly Sensitive Detection of the SARS-CoV-2 N Protein. Langmuir.

[146] Ma H., Wang Y., He L., Lian Z., Wang Y., Niu Y., Li N., Ye J., Ma Y. (2025). Platinum(II) complex with excellent electrochemiluminescence properties for the sensitive detection of glutathione and glutathione reductase activities. Microchem. J..

[147] Jiang H., Qin Z., Zheng Y., Liu L., Wang X. (2019). Aggregation-Induced Electrochemiluminescence by Metal-Binding Protein Responsive Hydrogel Scaffolds. Small.

[148] Zhang Y., Chen Y., Nie Y., Yang Z., Yuan R., Wang H., Chai Y. (2022). Highly Efficient Aggregation-Induced Electrochemiluminescence of Al(III)-Cbatpy Metal–Organic Gels Obtained by Ultrarapid Self-Assembly for a Biosensing Application. Anal. Chem..

[149] He Y., Shen W., Zhao J., Yang G., Yuan R., Chen S. (2024). Conjugated polymer-encapsulation-manipulated aggregation-induced electrochemiluminescence of tetraphenylethylene for sensitive malathion analysis. Sensor Actuat. B-Chem..

[150] Yoo H.S., Jeong S.H., Oh K.T., Lee S., Sohn Y.H., Ye B.S., Yun M., Lee P.H. (2022). Interrelation of striatal dopamine, brain metabolism and cognition in dementia with Lewy bodies. Brain.

[151] Frankle W.G., Himes M., Mason N.S., Mathis C.A., Narendran R. (2022). Prefrontal and Striatal Dopamine Release Are Inversely Correlated in Schizophrenia. Biol. Psychiat..

[152] Xie Z., Shao M., Liu Z., Ren X., Gao M., Ma H., Zhang N., Wei Q. (2024). Ultrasensitive aggregation-induced electrochemiluminescence sensor for dopamine detection in polymer hydrogel system. Sensor Actuat. B-Chem..

[153] Vasenko O., Zinchenko I., Tsytlishvili K., Bikasov V. (2020). Research of methods of inactivation of the antibiotic cyprofloxacin in order to prevent environmental pollution and protect human health. Sci. Horiz..

[154] Wang X.Y., Zhu K.D., Zhu J., Ding S.N. (2021). Photonic Crystal of Polystyrene Nanomembrane: Signal Amplification and Low Triggered Potential Electrochemiluminescence for Tetracycline Detection. Anal. Chem..

[155] Li S., Pang C., Ma X., Wu Y., Wang M., Xu Z., Luo J. (2022). Aggregation-induced electrochemiluminescence and molecularly imprinted polymer based sensor with Fe3O4@Pt nanoparticle amplification for ultrasensitive ciprofloxacin detection. Mircochem. J..

[156] Skog J., Würdinger T., van Rijn S., Meijer D.H., Gainche L., Curry W.T., Carter B.S., Krichevsky A.M., Breakefield X.O. (2008). Glioblastoma microvesicles transport RNA and proteins that promote tumour growth and provide diagnostic biomarkers. Nat. Cell Biol..

[157] Cassiday L.A., Maher L.J. (2001). In Vivo Recognition of an RNA Aptamer by Its Transcription Factor Target. Biochemistry.

[158] Jenison R.D., Gill S.C., Pardi A., Polisky B. (1994). High-Resolution Molecular Discrimination by RNA. Science.

[159] Lu L., Zhang L., Miao W., Wang X., Guo G. (2020). Aggregation-Induced Electrochemiluminescence of the Dichlorobis(1,10-phenanthroline)ruthenium(II) (Ru(phen)2Cl_2_)/Tri-n-propylamine (TPrA) System in H_2_O–MeCN Mixtures for Identification of Nucleic Acids. Anal. Chem..

[160] Zhao J., Tan X., He Y., Yuan R., Wang S., Chen S. (2024). Host–Guest Recognition-Mediated Supramolecular Aggregation-Induced Electrochemiluminescence of Iridium(III) Complexes for Nucleic Acid Bioassay. Anal. Chem..

[161] Liu J., Ming W., Zhang J., Zhou X., Qin Y., Wu L. (2024). Aggregation-induced electrochemiluminescence based on intramolecular charge transfer and twisted molecular conformation for label-free Immunoassay. Anal. Chim. Acta.

[162] Wang Y., Liu J., Niu Y., He L., Wang Y., Ma Y., Yao Y., Ye J. (2023). Tetraphenylethene-Based Multicomponent Platinum (II) Metallacages with Tunable Luminescence and Aggregation-Induced Electrochemiluminescence Properties. Adv. Opt. Mater..

[163] Jia D., Hua Y., Wu T., Ren X., Gao X., Yang L., Wei Q. (2025). Facile preparation of iridium-based AIE polymer dots for sensitive electrochemiluminescence immunoassay of CD44 protein. Anal. Chim. Acta.

[164] Li S., Li J., Geng B., Yang X., Song Z., Li Z., Ding B., Zhang J., Lin W., Yan M. (2021). TPE based electrochemiluminescence for ALP selective rapid one-step detection applied in vitro. Microchem. J..

[165] Yang L., Sun X., Wei D., Ju H., Du Y., Ma H., Wei Q. (2021). Aggregation-Induced Electrochemiluminescence Bioconjugates of Apoferritin-Encapsulated Iridium(III) Complexes for Biosensing Application. Anal. Chem..

[166] Guo J., Feng W., Du P., Zhang R., Liu J., Liu Y., Wang Z., Lu X. (2020). Aggregation-Induced Electrochemiluminescence of Tetraphenylbenzosilole Derivatives in an Aqueous Phase System for Ultrasensitive Detection of Hexavalent Chromium. Anal. Chem..

[167] Li S., Ma X., Pang C., Wang M., Yin G., Xu Z., Li J., Luo J. (2021). Novel chloramphenicol sensor based on aggregation-induced electrochemiluminescence and nanozyme amplification. Biosens. Bioelectron..

[168] Saremi M., Amini A., Heydari H. (2019). An aptasensor for troponin I based on the aggregation-induced electrochemiluminescence of nanoparticles prepared from a cyclometallated iridium(III) complex and poly(4-vinylpyridine-co-styrene) deposited on nitrogen-doped graphene. Microchim. Acta.

[169] Li J., Jia H., Ren X., Li Y., Liu L., Feng R., Ma H., Wei Q. (2022). Dumbbell Plate-Shaped AIEgen-Based Luminescent MOF with High Quantum Yield as Self-Enhanced ECL Tags: Mechanism Insights and Biosensing Application. Small.

[170] Yan M., Feng S., Yu L., Xue Y., Huang J., Yang X. (2021). Label-free immunosensor for cardiac troponin I detection based on aggregation-induced electrochemiluminescence of a distyrylarylene derivative. Biosens. Bioelectron..

[171] Xue J., Yang L., Du Y., Ren Y., Ren X., Ma H., Wu D., Ju H., Li Y., Wei Q. (2020). Electrochemiluminescence sensing platform based on functionalized poly-(styrene-co-maleicanhydride) nanocrystals and iron doped hydroxyapatite for CYFRA 21-1 immunoassay. Sensor Actuat. B-Chem..

[172] Hu C., Cao L., Wu X., Chen G., Li Y., Wang J., Huang C., Zhan L. (2024). Coreactant-free aggregation-induced electrochemiluminescence system based on the novel zinc-luminol metal-organic gel for ultrasensitive detection of PiRNA-823. Biosens. Bioelectron..

[173] Gao Y., Zhang L., Wang Z., Lu L. (2023). Aggregation-Induced Electrochemiluminescence and Nitric Oxide Recognition by Halogen Bonding with a Ruthenium(II) Complex. ChemPlusChem.

[174] Gao Y., Liu H., Li S., Xiao Y., Xiong C., Wen W., Wang S., Zhang X., Chen M.-M. (2025). Coordinative interaction-enhanced aggregation-induced electrochemiluminescence signal enables ultrasensitive aflatoxin B1 sensing in corn. Food Chem..

[175] Chen J.-J., Qin N., Yuan R., Wang H.-J. (2025). Solvent Regulation Enhanced Aggregation-Induced Electrochemiluminescence of Piperazine-Functionalized Carbon Dot Aggregates for Ultrasensitive Detection of Deoxynivalenol. Anal. Chem..

[176] Chen J.-J., Pan M.-Q., Cao W.-W., Wang Z., Yuan R., Wang H.-J. (2024). Solvent Regulation Induced Cathode Aggregation-Induced Electrochemiluminescence of Tetraphenylethylene Nanoaggregates for Ultrasensitive Zearalenone Analysis. Anal. Chem..

[177] Chen X., Zhao J., Wang Y., Yuan R., Chen S. (2024). Dual emitting aggregation-induced electrochemiluminescence from tetrastyrene derivative for chloramphenicol detection. Food Chem..

[178] Yi J., Sun Y., Wang X., Du Y., Feng R., Dong X., Liu X., Ma H., Wei Q. (2025). Protein-confinement synergizes with aggregation-induced-emission enhancement strategy for tetracycline detection. Sensor Actuat. B-Chem..

[179] Zhou X., Song H., Zhang X., Chen L., Chen Y., Li Z., Feng L. (2025). Aggregation-induced Electrochemiluminescence of AgNCs Enhanced with AuNPs@MXene Composites for Ultrasensitive Detection of microRNA. Chem-Asian J..

[180] Yang J.-L., Wang L., Chen Y.-F., Wang Z., Yuan R., Wang H.-J. (2025). Efficient Al–H3NTB-MOG ECL Emitter with Self-Enhanced and AIECL Performance for Ultrasensitive Sensing of miRNA-141 Combined with a Y-Shaped Multiregion Dual-Drive DNA Walker. Anal. Chem. Anal. Chem..

